# Hypoosmolarity inhibits ammonia oxidation by terrestrial and freshwater *Nitrosopumilaceae* members

**DOI:** 10.1093/ismejo/wrag045

**Published:** 2026-03-06

**Authors:** Joo-Han Gwak, Adebisi Olabisi, Ui-Ju Lee, Christiana Abiola, Seongjun Lee, Hackwon Do, Yun Ji Choi, Jay-Jung Lee, Man-Young Jung, Nico Jehmlich, Martin von Bergen, Michael Wagner, Samuel Imisi Awala, Zhe-Xue Quan, Sung-Keun Rhee

**Affiliations:** Department of Life Science, Hallym University, Chuncheon 24252, Gangwon-do, Republic of Korea; Multidisciplinary Genome Institute, Hallym University, Chuncheon 24252, Gangwon-do, Republic of Korea; Department of Biological Sciences and Biotechnology, Chungbuk National University , Cheongju 28644, Chungcheongbuk-do, Republic of Korea; Department of Biological Sciences and Biotechnology, Chungbuk National University , Cheongju 28644, Chungcheongbuk-do, Republic of Korea; Department of Biological Sciences and Biotechnology, Chungbuk National University , Cheongju 28644, Chungcheongbuk-do, Republic of Korea; Department of Biological Sciences and Biotechnology, Chungbuk National University , Cheongju 28644, Chungcheongbuk-do, Republic of Korea; Division of Life Sciences, Korea Polar Research Institute, Incheon 21990, Republic of Korea; Interdisciplinary Graduate Program in Advanced Convergence Technology and Science, Jeju National University, Jeju 63243, Jeju-do, Republic of Korea; Geum River Environment Research Center, National Institute of Environmental Research, Okcheon 29027, Chungcheongbuk-do, Republic of Korea; Interdisciplinary Graduate Program in Advanced Convergence Technology and Science, Jeju National University, Jeju 63243, Jeju-do, Republic of Korea; Department of Biology Education, Jeju National University, Jeju 63243, Jeju-do, Republic of Korea; Department of Molecular Systems Biology, Helmholtz Centre for Environmental Research–Zentrum für Umweltforschung GmbH, Leipzig 04318, Germany; Department of Molecular Systems Biology, Helmholtz Centre for Environmental Research–Zentrum für Umweltforschung GmbH, Leipzig 04318, Germany; Faculty of Biosciences, Pharmacy and Psychology, Institute of Biochemistry, University of Leipzig, Leipzig 04103, Germany; Centre for Microbiology and Environmental Systems Science, Department of Microbiology and Ecosystem Science, University of Vienna, 1090 Vienna, Austria; Department of Chemistry and Bioscience, Aalborg University, 9220 Aalborg, Denmark; Department of Biological Sciences, University of Calgary, Calgary, AB T2N 1N4, Canada; School of Life Sciences, Fudan University, Shanghai 200438, China; Department of Biological Sciences and Biotechnology, Chungbuk National University , Cheongju 28644, Chungcheongbuk-do, Republic of Korea

**Keywords:** hypoosmolarity, nitrification, soil and freshwater ecosystems, ammonia-oxidizing archaea

## Abstract

Salinity strongly influences the physiology and distribution of nitrifying microorganisms, yet the effects of low salinity remain understudied. This study investigates the impact of hypoosmolarity on different groups of ammonia oxidizers in soil and freshwater reservoirs, as well as in pure culture isolates. In soil microcosms amended with ammonium, at low salinity levels (~120 μS/cm), comparable to values commonly found in pristine terrestrial and freshwater environments, the abundance of ammonia-oxidizing bacteria (AOB), dominated by *Nitrosomonas oligotropha*, significantly increased. In contrast, the growth of ammonia-oxidizing archaea (AOA), dominated by “*Candidatus* Nitrosotenuis” of the *Nitrosopumilaceae* family, was stimulated by high salinity (~760 μS/cm). In ammonium-fed freshwater microcosms, the abundance of AOB, dominated by *N. oligotropha,* significantly increased under both low (~170 μS/cm) and high salinity (~850 μS/cm) conditions. In the presence of allylthiourea (50 μM), used to inhibit bacterial ammonia oxidation, AOA were sensitive to low salinity in both soil and freshwater microcosms. Consistently, culture-dependent studies revealed marked growth inhibition of terrestrial AOA, especially members of *Nitrosopumilaceae*, under hypoosmolarity, unlike AOB and complete ammonia oxidizer (comammox) strains. Genomic analyses, along with transcriptomic studies, suggested that the sensitivity of AOA to hypoosmolarity stress was possibly due to a lack of osmoregulatory transport systems and their S-layer cell wall structure. Overall, this study indicates hypoosmolarity as an important factor shaping the ecological niches and distribution of ammonia oxidizers, as well as nitrification activities, in terrestrial and freshwater environments that are increasingly affected by intensified water cycles due to global change.

## Introduction

Ammonia oxidation is the first and often rate-limiting step of nitrification in the nitrogen cycle [1]. This process is mostly driven by chemolithoautotrophic ammonia oxidizers, which conserve energy by oxidizing ammonia (NH_3_) to either nitrite (NO_2_^−^) or nitrate (NO_3_^−^) [1–3]. As both climatic and anthropogenic drivers alter regional water cycles [4, 5], fluctuations in salinity across terrestrial and freshwater environments are becoming more pronounced due to shifts in precipitation patterns and more frequent extreme weather events such as flooding and drought [6, 7]. These changes, in turn, alter microbial habitats by disrupting osmotic balance and ion homeostasis, both of which are critical for the stability of microbial communities that mediate the nitrification process [8–10]. Despite the widespread occurrence of hypoosmotic conditions in terrestrial and freshwater environments [11, 12], little is known about how microorganisms, particularly ammonia oxidizers, adapt to chronic hypoosmotic stress. Investigating their responses under environmentally relevant salinity conditions is essential for predicting shifts in nitrogen cycling in terrestrial and freshwater ecosystems influenced by global water dynamics.

Climate change, driven by global warming, is significantly intensifying the Earth’s water cycle [4, 5]. This intensification leads to contrasting hydrological extremes, including localized desertification and frequent flooding [6, 7]. Consequently, salinity trends exhibit high regional variability driven by both climatic and anthropogenic factors. In arid and semi-arid regions, water overuse and intensive irrigation, combined with high evaporation, drive severe salinization in soils and shrinking lakes, as famously exemplified by the Aral Sea crisis [13]. However, in contrast to these salinization trends, projections indicate that by 2100, many regions will experience a marked increase in heavy precipitation events [14], which will substantially alter hydrologic dynamics toward dilution. These precipitation changes are likely to have far-reaching effects on various environmental parameters, particularly salinity. Whereas salinization is a critical issue in drylands, increased precipitation in temperate and tropical zones tends to dilute and thus decrease the salinity of soil pore waters, surface waters (such as lake/reservoirs and rivers), and groundwater in aquifers. Therefore, understanding ecosystem responses to hypoosmolarity is as crucial as understanding responses to salinization.

Shifts in salinity are key abiotic factors influencing microbial physiology. The normal functioning of microbial cells relies heavily on the regulation of intracellular ion concentrations [15]. Osmoregulation, the process by which cells maintain water and salt balance, requires considerable energy and is fundamental for cellular survival under both hyperosmotic and hypoosmotic stress conditions. Consequently, variations in salinity can significantly influence the physiology and ecology of microorganisms [16], as well as other macroorganisms inhabiting terrestrial and freshwater ecosystems. These shifts in microbial physiology, driven by changes in salinity, may subsequently impact key biogeochemical processes, potentially disrupting nutrient cycling, modifying microbial production and consumption of greenhouse gasses, and altering overall ecosystem function.

Nitrification, the sequential aerobic oxidation of reduced nitrogen compounds, primarily NH_3_ to NO_3_^−^ via NO_2_^−^, is considered pivotal in the global biogeochemical nitrogen cycle on Earth. Furthermore, ammonia oxidation is a key process that contributes to the release of nitrous oxide (N_2_O), a potent greenhouse gas and ozone-depleting substance [17]. This process is microbially executed by different yet co-occurring groups of chemolithoautotrophic ammonia oxidizers, specifically ammonia-oxidizing archaea (AOA) and ammonia-oxidizing bacteria (AOB), which can oxidize NH_3_ to NO_2_^−^ using it as their sole energy-conserving substrate [1, 18]. Additionally, a group of bacterial ammonia oxidizers that perform complete ammonia oxidation (comammox; CMX) of NH_3_ to NO_3_^−^, was discovered [2, 3]. This group consists of clades A and B, which exhibit different distributions depending on habitat [19]. AOA, AOB, and CMX differ in their kinetic properties and N_2_O-yields [20–24], which likely contribute to their distinct niche partitioning across habitats. Nevertheless, ammonia-oxidizing communities in terrestrial and freshwater environments exhibit remarkable variability, with compositions that remain largely unpredictable despite our growing understanding of key environmental drivers such as pH, temperature, moisture, and nutrient availability [25–30].

Previous studies on the impact of salinity on nitrification have mostly focused on hyperosmolarity, as high salinity is often a serious concern in agricultural and environmental contexts [8, 10, 31–35]. In contrast, salinity below 350 μS/cm has been associated with reduced microbial diversity in a freshwater microcosm study [36], suggesting this level as an ecologically meaningful threshold for defining hypoosmotic stress [37, 38]. In pristine environments, salinity—typically assessed via electrical conductivity (EC)—is often well below this threshold. In lake/reservoirs, the 25th percentile EC is ~100 μS/cm, the median is 227 μS/cm, and the 75th percentile is 427 μS/cm (Supplementary Fig. S1) [11], highlighting the prevalence of hypoosmotic conditions. Similarly, in soils, EC values derived from soil water extracts show the 25th percentile of 100 μS/cm (based on imputed data due to sparse EC measurements in the dataset), the median of 130 μS/cm, and the 75th percentile of 410 μS/cm (Supplementary Fig. S1) [12]. These salinity levels are substantially lower than those of typical laboratory mineral media [21, 39–41], such as the artificial freshwater medium (AFM; ~3600 μS/cm) [35], which is commonly used to cultivate and study various microorganisms, including soil and freshwater ammonia oxidizers.

Investigating the impact of environmentally relevant hypoosmotic conditions on ammonia oxidizer activity is vital for understanding current nitrogen cycling processes and predicting future changes in nitrogen cycling dynamics due to shifts in global water cycles [42, 43]. Although most laboratory studies on hypoosmolarity have focused on the microbial cell responses to rapid and acute decreases in salinity (i.e. hypoosmotic shock) under laboratory conditions [44–47], few have examined the adaptation of microorganisms to “chronic” hypoosmotic stress [48–51]. In this study, we investigated the effect of chronic hypoosmolarity on ammonia oxidizer activity in soils and freshwater environments. The activity of AOA, particularly those within the *Nitrosopumilaceae* family (hereafter referred to as AOA-NpF) [52], was sensitive to hypoosmolarity, a finding further supported by pure culture experiments. Overall, we propose that hypoosmolarity serves as an important environmental factor contributing to the niche differentiation and abundance of ammonia oxidizers in terrestrial and freshwater environments.

## Materials and methods

### Environmental salinity and media composition

EC values (μS/cm), as a measure of salinity, were obtained from global datasets of lake/reservoirs [11] and soils [12] to characterize typical environmental salinity ranges. To ensure relevance, the datasets were filtered to include only non-coastal lake/reservoirs and soils with non-zero EC values, as zero entries may reflect conditional imputation in the original dataset [12], and percentile statistics were interpreted accordingly. Subsequently, EC percentiles were calculated to identify representative salinity levels typically observed in these environments. In combination with data on natural freshwater salt composition [53], these EC values informed the design of a mineral water medium (MWM) intended to mimic the ionic compositions of natural freshwaters (Supplementary Table S1). Varying salinity levels were achieved by preparing different strengths of MWM through the adjustment of monovalent and divalent cation concentrations.

For microcosm experiments, NH_4_Cl was used as a substrate. KH_2_PO_4_, NaHCO_3_, HEPES, pyruvic acid, trace elements, and ferric sodium EDTA were added as supplements depending on experimental conditions, with concentrations detailed in Supplementary Table S1. After all supplements were added, the EC of 1× MWM was measured at 128 μS/cm, corresponding to the lower EC range observed in lake/reservoirs waters and soil environments. In contrast, 10× MWM had an EC of 795 μS/cm, representing the higher EC range observed in lentic waters and soils, and was used for experiments requiring elevated salinity conditions (Supplementary Fig. S1). MWM was used to examine the effects of salinity on ammonia oxidizers in both microcosm and pure culture experiments. AFM [54], previously described and with an EC of 3540 μS/cm, served as a control.

### Microcosm experiments

Soil samples (top 30 cm) were collected from grassland and agricultural fields. The agricultural field was fertilized annually with oil cake (N:P:K = 4:2.1:1; 70% organic matter content) and potassium chloride, providing ~190 kg N ha^−1^ year^−1^, 48.8 kg P ha^−1^ year^−1^, and 123.7 kg K ha^−1^ year^−1^, split into three applications from May to September. Metadata, including sampling location, date, depth, EC, pH, and nutrient levels, are provided in Supplementary Table S2. The samples were sieved through a 4-mm mesh and stored at 4°C until use. Soil slurry microcosms were set up in 250-ml polystyrene culture flasks, each containing 3 g of fresh soil mixed with 150 ml of 0×, 1×, or 10× MWM, with triplicate samples for each condition. The intrinsic salinity contribution from soil samples was negligible. For the initial sample, 10 g of fresh soil were prepared separately and used for DNA extraction. Freshwater samples were collected at 20 m depth, corresponding to the beginning of the aphotic zone, from water reservoirs Soyang and Daecheong, with sampling details provided in Supplementary Table S2. Freshwater microcosms were prepared using 150 ml of unfiltered freshly sampled water alone or supplemented with 10× MWM basic salts, in triplicate for each condition. For the initial sample, 1 l of reservoir water was filtered separately through 0.1-μm pore-size mixed cellulose ester filters (Advantec, Japan), and the filters were used for DNA extraction.

NH_4_Cl (100 μM or 25 μM) was added as a substrate to the soil and freshwater microcosms, respectively. All microcosms were incubated at 25°C in the dark with intermittent inversion. Ammonia, nitrite, and nitrate concentrations were measured to monitor nitrification activity, following previously described methods [54]. To specifically monitor nitrification by AOA, a control microcosm was treated with allylthiourea (ATU; 50 μM) to selectively inhibit AOB and CMX [2, 55]. Once ammonium oxidation was complete, all microcosms (>140 ml) were harvested by filtration using 0.1-μm pore-size mixed cellulose ester filters and stored at −80°C before DNA extraction.

### DNA extraction, qPCR, and 16S rRNA gene amplicon sequencing

Genomic DNA was extracted from filters that had been ground in liquid nitrogen using the Soil DNA Kit (GeneAll, Korea) according to the manufacturer’s instructions. DNA concentration and integrity were assessed using a Qubit 4 Fluorometer (Thermo Fisher Scientific, USA) and by agarose gel electrophoresis. Extracted DNA samples were stored at −80°C for downstream applications.

Quantitative PCR (qPCR) was conducted using specific primers targeting AOA, AOB, and CMX groups, with sequences and conditions provided in Supplementary Table S3. Standard curves generated in each reaction were linear. For all assays (AOA, AOB, CMX-A, and CMX-B), standard curves were constructed using 10^2^–10^8^ copies. AOA standards consisted of a genomic DNA mixture of *Nitrosarchaeum koreense* MY1 and *Nitrososphaera viennensis* EN76, AOB standards used genomic DNA from *Nitrosomonas europaea* ATCC 19718, and CMX standards were prepared as previously described [56]. All assays showed amplification efficiencies of 92%–96% for AOA, 85%–97% for AOB, 86%–92% for CMX-A, and 88%–91% for CMX-B, with *R*^2^ values >0.98. The detection limit of each qPCR assay was defined as the lowest standard concentration that consistently yielded positive amplification (10^2^ copies per reaction). After each run, qPCR specificity was confirmed by melting-curve analysis, and amplicons were checked on 2.5% agarose gels to exclude nonspecific products. To assess potential PCR inhibition, dilution-series tests of DNA samples were performed, with no indication of partial PCR inhibition.

The hypervariable V4–V5 region of the 16S rRNA gene was amplified using the primer pair 515F/926R, along with sample-specific indexing adapters (Nextera XT Index Kit), following the manufacturer’s instructions. The amplification consisted of an initial denaturation at 95°C for 3 min, followed by 25 cycles of 95°C for 45 s, 50°C for 45 s, and 72°C for 90 s, and a final extension at 72°C for 5 min. Amplicons were purified using the Labopass purification kit (Cosmo Genetech, South Korea), and their concentration and quality were assessed as described above. Sequencing was performed on the MiSeq System (Illumina) with 300-bp paired-end reads by LabGenomics Inc., Korea. Raw sequence reads were quality-checked using FastQC (v0.11.8) [57], and adapter trimming and quality filtering were performed using Cutadapt (v4.9) [58], as integrated into the QIIME2 pipeline (qiime2-amplicon-2024.10) [59]. Reads were demultiplexed in QIIME2 and processed to generate amplicon sequence variants (ASVs) using the DADA2 package (v1.20.0) [60] within R. ASVs associated with chloroplasts, mitochondria, and singletons were filtered out before downstream analysis. Taxonomic classification of ASVs was performed using the Greengenes2 database (2022.10) [61]. Building on this classification, phylogenetic analysis was further conducted to refine the ammonia oxidizers, including the *Nitrospira* lineage.

### Phylogenetic analysis

ASVs of 16S rRNA genes were aligned using MAFFT (v7.526) [62] with L-INS-i algorithm based on structurally curated seed alignments of bacterial and archaeal 16S rRNA gene sequences retrieved from the Comparative RNA Website (as of July 2024) [63]. Phylogenetic relationships were inferred using the maximum-likelihood method implemented in IQ-TREE (v2.3.6) [64], with ModelFinder (−m MFP) [65]. The phylogenetic tree was visualized using iTOL (v6.9.1) [66].

### Effect of hypoosmotic stress on ammonia oxidizer strains

The effect of hypoosmotic stress on growth was tested across a range of salinity levels (0–128× MWM) with four AOA strains, two AOB strains, one AOB enrichment culture, and one CMX strain. The four AOA strains included two from AOA-NpF: *N. koreense* MY1, isolated from agricultural soil in Korea [67], and “*Candidatus* Nitrosotenuis chungbukensis” MY2, isolated from a deep oligotrophic soil horizon [68]; and two from the *Nitrososphaeraceae* family (hereafter referred to as AOA-NsF): *N. viennensis* EN76, isolated from garden soil [69], and “*Candidatus* Nitrosocosmicus oleophilus” MY3, isolated from coal tar-contaminated sediment [70]. The AOB cultures included *N. europaea* ATCC 19718 from the *N. europaea* lineage, isolated from soil [71]; an enrichment culture from Daecheong reservoir microcosm experiments, dominated by the *Nitrosomonas oligotropha* lineage (Supplementary Dataset S1), designated as the LD1 enrichment culture, established by serial dilution under 0× MWM conditions supplemented with 0.1 mM NH_4_Cl; and *Nitrosomonas nitrosa* Nm90 from the *N. communis* lineage, isolated from industrial sewage [72]. The CMX strain was, “*Candidatus* Nitrospira inopinata” ENR4, isolated from a biofilm in a hot-water pipe of an oil-exploration well [3]. For *N. europaea* ATCC 19718, *N. nitrosa* Nm90, and “*Ca.* Nitrospira inopinata” ENR4, 0.5 mM NH_4_Cl was used; in contrast, 0.1 mM NH_4_Cl was used for all other strains or the LD1 enrichment culture, with the specific growth rate (μ) determined based on NH_4_^+^ oxidation. Cultures were incubated under oxic conditions with ambient air, without shaking, and in the dark. The incubation temperatures were set to 25°C for *N. koreense* MY1, *N. europaea* ATCC 19718, and the LD1 enrichment culture; 28°C for *N. nitrosa* Nm90; 30°C for “*Ca*. N. chungbukensis” MY2 and “*Ca*. N. oleophilus” MY3; 37°C for “*Ca.* N. inopinata” ENR4; and 42°C for *N. viennensis* EN76. The specific growth rate (μ) was calculated from three to five time points during the exponential growth phase by determining the slope of the log-transformed nitrite concentration over time, using the equation μ = (ln*N*_1_ − ln*N*_0_)/(*t*_1_ − *t*_0_), where (ln*N*_1_ − ln*N*_0_) represents the change in the natural logarithm of nitrite concentration, and (*t*_1_ − *t*_0_) represents the change in time. The time point of complete ammonium oxidation under the 10× MWM condition served as a reference for incubation timing, and this condition was further incubated for several additional days after complete oxidation. All cultures were monitored sufficiently beyond this reference to ensure reliable determination of growth rates under each condition.

### Statistical analysis

To evaluate differences in ammonia oxidizer abundance under different microcosm conditions, qPCR-derived abundance data were analyzed using one-way analysis of variance (ANOVA) performed separately for each ammonia oxidizer group. Homogeneity of variance was assessed using the Brown–Forsythe test. When unequal variances were detected, Welch’s ANOVA followed by Dunnett’s T3 test was applied. Differences were considered statistically significant at *P* < .05. Statistical analyses were conducted using GraphPad Prism (v10.6.1).

For 16S rRNA gene amplicon sequencing data, differential abundance analysis was performed using the DESeq2 package (v1.40.2) [73] in R (v4.3.2) [74] based on raw sequencing count data. Resulting *P* values were adjusted for multiple testing using the Benjamini–Hochberg procedure, and taxa with adjusted *P* < .05 were considered statistically significant.

#### Comparative genomic analysis

To investigate the genomic repertoire of osmoregulatory systems in ammonia oxidizers, a total of 463 high-quality genomes (completeness >90%, contamination <10%, assessed by CheckM [75]) were retrieved from the NCBI database (Supplementary Dataset S2), including 266 AOA, 179 AOB, and 18 CMX genomes. The presence or absence of osmoregulatory genes was determined based on KEGG Orthology (KO) annotations using KofamScan [76] and BlastKOALA [77], both with default parameters. Phylogenomic relationships among genomes were inferred following the Anvi’o phylogenomics workflow [78], using IQ-TREE (v2.3.6) [64] with the LG+G+F model. The resulting phylogenomic tree was visualized in iTOL (v6.9.1) [66].

#### Transcriptomic and proteomic analysis

To assess the differential expression of genes under varying salinity conditions, 1× and 10× MWM—representing low and high salinity in environments, respectively—were used. Representative AOA strains from AOA-NpF (“*Ca.* N. chungbukensis” MY2) and AOA-NsF (*N. viennensis* EN76), as well as the AOB strain *N. europaea* ATCC 19718 were prepared and inoculated into 2-L glass bottles containing 1 l of medium, with six replicates for each salinity condition. The cultures were incubated at 25°C for “*Ca*. N. chungbukensis” MY2 and *N. europaea* ATCC 19718, and at 42°C for *N. viennensis* EN76, respectively. During the mid-exponential growth phase, when ~70 μM of the initial 100 μM ammonium had been oxidized, each culture was filtered through a 0.1-μm pore size mixed cellulose ester filter. Filters were immediately flash-frozen in liquid nitrogen and stored at −80°C.

Total nucleic acids were extracted from filters that had been ground in liquid nitrogen, following the manual extraction procedure described previously [79]. The resulting lysates were further processed using the AllPrep DNA/RNA Mini Kit (Qiagen, Germany) to purify and separate RNA and DNA. DNase treatment was included during RNA purification to remove residual genomic DNA. RNA integrity was assessed using a TapeStation RNA ScreenTape (Agilent, USA). cDNA libraries were constructed using the Stranded Total RNA Library Prep with Ribo-Zero Plus kit (Illumina, USA) and sequenced on an Illumina NovaSeqX platform as paired-end reads (2 × 150 bp) by Phyzen Inc., Korea. Quality control of the sequencing reads was conducted using FastQC (v0.11.8) [57], and adaptor and quality trimming were performed using BBTools (v39.06) [80]. For downstream transcriptomic and proteomic analyses, genomes were obtained from the following sources: “*Ca.* N. chungbukensis” MY2 (10.6084/m9.figshare.30947483), *N. viennensis* EN76 (NCBI accession CP007536.1), and *N. europaea* ATCC 19718 (NCBI accession NC_004757.1). Reads mapping to rRNA sequences were removed using SortMeRNA (v2.1) [81]. The remaining reads were then aligned to the genomes using STAR (v2.7.11a) [82], and the mapped reads for each gene were counted using HTSeq (v2.0.5) [83]. A summary of the sequencing results is provided in Supplementary Dataset S3. Gene expression levels were calculated and presented as transcripts per kilobase million. Differential expression analysis was conducted using the DESeq2 package (v1.40.2) [73] in R (v4.3.2) [74]. *P* values were calculated using a two-sided Wald test, and multiple comparisons were adjusted using the Benjamini–Hochberg method, as implemented in the DESeq2 package.

For proteomic analysis, proteins were extracted from the same filtered biomass as used for RNA extraction. The filter was transferred into a 1.5-ml screw-cap tube, followed by the addition of three zirconia beads and three glass beads. Subsequently, 500 μl of UT buffer (6 M urea / 4 M thiourea) was added, and the samples were incubated for 10 min at 70°C with shaking at 1400 rpm. Cell disruption was performed using three FastPrep cycles (5.5 m/s, 60 s each). The lysates were then centrifuged at full speed for 10 min at 4°C, and proteins were precipitated overnight with trichloroacetic acid (TCA, final concentration 25%) at 4°C. After precipitation, samples were centrifuged again at full speed for 10 min at 4°C, and the resulting protein pellets were washed with acetone and air-dried. Proteins were briefly separated by SDS-PAGE, running only until the samples entered the separating gel, followed by Coomassie staining. The entire protein band from each sample was excised and cut into small pieces, which were subjected to overnight digestion with Lys-C and trypsin. Peptides were desalted using SOLA μ columns, the resulting peptide pellets were dissolved in 0.1% formic acid, and concentrations were measured using a Nanodrop spectrophotometer to adjust all samples to 0.2 μg/μl. Following protein extraction and enzymatic digestion with trypsin, peptide mixtures were analyzed using a nano-flow high-performance liquid chromatography system (UltiMate 3000 RSLCnano, Dionex, Thermo Fisher Scientific). Peptides were first loaded onto a C18 trap column (PepMap100, 300 μm × 2 cm, 5 μm particle size, nanoViper, Thermo Fisher Scientific), and subsequently separated on a C18-reverse-phase analytical column (Acclaim PepMap 100, 75 μm × 25 cm, 3 μm particle size, nanoViper, Thermo Fisher Scientific). The separated peptides were introduced into a Q Exactive HF mass spectrometer (Thermo Fisher Scientific) coupled with a TriVersa NanoMate nano-electrospray ionization source (Advion, Harlow, UK) operating in LC chip coupling mode. Mass spectrometry acquisition parameters, including the LC gradient, ionization settings, and scan modes, followed previously established protocols [84].

Although proteomic analysis of the two AOA strains under different salinity conditions enabled the detection of some proteins corresponding to highly expressed genes identified in the transcriptomic analysis, detailed functional interpretation was limited by the proteome coverage.

## Results and discussion

### Responses of soil ammonia oxidizers to low salinity

**Figure 1:**
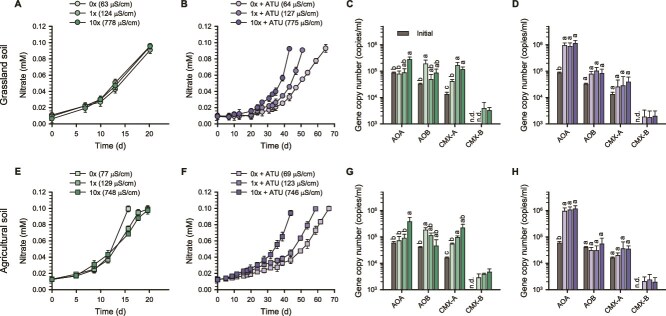
Changes in nitrate concentrations and the abundances of ammonia oxidizers during incubation of soil slurry microcosms. Results from microcosms using grassland soil (A–D) and agricultural soil (E–H), supplemented with 100 μM ammonia, are shown. Details of the microcosm setup are described in the Methods section. Nitrate concentrations (A, B, E, F) and the corresponding qPCR results (C, D, G, H) are presented. Microcosm samples were collected at the endpoint of ammonia oxidation (A, B, E, F) for downstream qPCR and 16S rRNA gene amplicon analysis. Panels B, F, D, and H show results from ATU-treated microcosms. Error bars represent standard deviations of biological replicates (*n* ≥ 3). Different letters denote statistically significant differences among treatments, including the initial time point. Statistical analyses were performed separately for each ammonia oxidizer group as described in the Methods section (*P* < .05). n.d., not detected.

The effects of varying salinity levels (with ECs ranging from 60 to 780 μS/cm) on nitrification activity and the ammonia-oxidizing community were investigated using soil slurry microcosms derived from grassland and agricultural soils. The ECs of the soil slurry microcosms prepared with 0× (without basic salts; EC ~60 μS/cm) and 1× MWM (EC ~120 μS/cm) were comparable to those observed in low-salinity soil environments, aligning with the 50th percentile of soil ECs (Fig. 1; Supplementary Fig. S1) [12]. For comparison, soil slurry microcosms prepared with 10× MWM had an EC value of ~780 μS/cm, corresponding to the 90th percentile of soil ECs. Microcosms were further amended with or without ATU (50 μM), an inhibitor specific for bacterial ammonia oxidation by AOB and CMX [2, 55], to differentiate the response of AOA from that of bacterial ammonia oxidizers. Activities of ammonia oxidizers in microcosms were assessed through the measurement of nitrate production from nitrification and changes in the abundances of AOA, AOB, and CMX following ammonia oxidation (Fig. 1).

Nitrate production rates were comparable across all salinity levels in both soil microcosms during the 20-day incubation (Fig. 1A and E). However, salinity-dependent differences in the abundances of *amoA* genes affiliated with distinct ammonia oxidizer groups became evident after incubation. The abundance of the AOA *amoA* gene increased significantly only under the 10× MWM conditions, whereas that of CMX-A gradually increased with rising salinity (*P* < .05; Fig. 1C and G). In contrast, the abundance of the AOB *amoA* gene was highest under 0× MWM conditions in both soils, displaying a trend opposite to that of CMX-A and AOA (Fig. 1C and G). The abundance of the CMX-B *amoA* gene remained a minor component, with no significant differences observed among salinity levels after incubation. Overall, although ammonia oxidation rates were comparable among salinity conditions (Fig. 1A and E), the growth responses of ammonia oxidizers varied: AOB was more abundant under lower salinity, whereas AOA and CMX-A became more prominent under higher salinity (Fig. 1C and G).

The response of AOA to salinity was further examined using ATU. Ammonia oxidation was delayed in the presence of ATU in both soil microcosms (Fig. 1B and F) compared to the control without ATU (Fig. 1A and E). AOA-specific growth rates, calculated based on nitrate production [54], were lower under 0× and 1× MWM conditions (0.015 d^−1^ and 0.020 d^−1^, respectively, in both soils) compared to 10× MWM conditions (0.027 d^−1^ and 0.032 d^−1^ in grassland and agricultural soils, respectively), indicating that AOA growth is sensitive to lower salinity (Fig. 1B and F). This trend is consistent with the observed higher abundance of AOA at higher salinity in the absence of ATU (Fig. 1C and G). As expected, AOA were more abundant than AOB and CMX (Fig. 1D and H) across all salinity conditions in both soils, likely due to the inhibitory effect of ATU on bacterial ammonia oxidation. Nevertheless, AOB and CMX-A *amoA* gene copies showed minor increases under ATU treatment (Fig. 1D and H), indicating that inhibition was not complete or may have weakened during the incubation period, possibly due to degradation or inactivation of ATU within the microcosm [85].

### Soil ammonia-oxidizing community analysis

**Figure 2:**
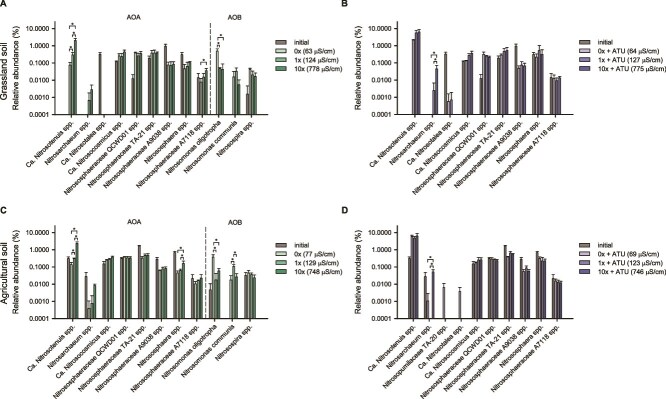
Relative abundances of ammonia oxidizers in soil slurry microcosm samples. The relative abundances (as % of total 16S rRNA gene reads) of ammonia oxidizers at the genus level are shown for microcosms derived from grassland soil (A, B) and agricultural soil (C, D). Panels B and D show results from the ATU-treated microcosms. Taxa detected in only a single replicate across all treatments or with abundances below 0.002% were excluded from the analysis. Error bars represent standard deviations of biological replicates (*n* ≥ 3). Initial samples were excluded from statistical analyses. Asterisks indicate statistically significant differences among treatments based on DESeq2 analysis of raw count data (adjusted *P* ≤ .05). ASV-level information for all ammonia oxidizers detected, including AOB and *Nitrospira* in ATU-treated microcosms, is provided in Supplementary Dataset S1. Species-level differential abundance results based on DESeq2 are provided in Supplementary Dataset S4.

To complement the qPCR results from the soil slurry microcosm experiments, 16S rRNA gene amplicon sequencing was conducted to analyze the ammonia-oxidizing community in samples collected at the initial time point and after the oxidation of added ammonia was complete (Fig. 2). In the absence of ATU, the relative abundance of the genus “*Ca*. Nitrosotenuis” within AOA-NpF significantly increased in response to elevated salinity in both soils (*P* < .05; Fig. 2A and C; Supplementary Dataset S4). This increase was primarily driven by ASV1979, which on average comprised 93% of the reads assigned to “*Ca*. Nitrosotenuis” in each sample and is identical in sequence to the corresponding 16S rRNA gene region of “*Ca.* N. chungbukensis” MY2 (Supplementary Fig. S2A; Dataset S1). The relative abundance of the genus *Nitrososphaera* within AOA-NsF in agricultural soil was also significantly higher under 10× MWM than under 0× MWM (Fig. 2C). In contrast, members of the *N. oligotropha* lineage within AOB were most abundant under lower salinity conditions, showing a significant response under those conditions in both soils (*P* < .05; Fig. 2A and C; Supplementary Dataset S4). ASV53, which is identical in sequence to *N. oligotropha* Nm45, was the dominant variant within this lineage, accounting for an average of 52% of the reads across samples (Supplementary Fig. S2B; Dataset S1). In agricultural soil, *Nitrosomonas communis* within AOB showed the highest relative abundance under 1× MWM, replacing the *N. oligotropha* lineage, which showed the highest relative abundance under 0× MWM (Fig. 2C). At 10× MWM, both lineages exhibited lower relative abundances than at their respective peaks under lower salinity conditions (Fig. 2A and C). Most of the *Nitrospira* reads belonged to lineage II, which was consistently detected in both soil types at relative abundances ranging from 0.7% to 4.1%, regardless of the salinity (Supplementary Fig. S2C; Dataset S1). Due to limitations in 16S rRNA gene resolution, CMX and nitrite-oxidizing bacteria within the genus *Nitrospira* are not discernible from each other [2, 3, 86] and were therefore excluded from this analysis. In the presence of ATU, the relative abundance of the genus “*Ca*. Nitrosotenuis” markedly increased in both soil microcosms following ammonia oxidation, dominating over other ammonia oxidizers regardless of salinity levels (*P* < .05; Fig. 2B and D; Supplementary Dataset S4), despite lower AOA specific growth rates under 0× and 1× MWM conditions compared to 10× MWM conditions (Fig. 1B and F). The same ASV1979, which was the predominant variant under ATU-free conditions, remained predominant in the presence of ATU (Supplementary Fig. S2A; Dataset S1).

### Responses of freshwater ammonia oxidizers to salinity change

**Figure 3:**
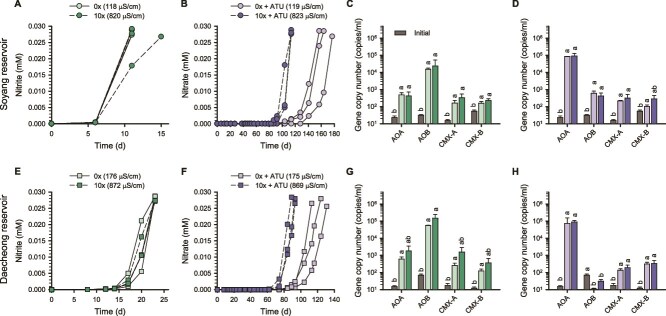
Changes in nitrite/nitrate concentrations and the abundances of ammonia oxidizers during incubation of freshwater microcosms. Results from microcosms using unfiltered natural water samples from Soyang reservoir (A–D) and Daecheong reservoir (E–H), supplemented with 25 μM ammonia, are shown. Details of the microcosm setup are described in the Methods section. In the absence of ATU, nitrite rather than nitrate accumulated (A, E). Each line represents an individual biological replicate, as high inter-replicate variability precluded meaningful averaging. Some lines overlap due to similar values (A, B, E, F). Microcosm samples were collected at the endpoint of ammonia oxidation (A, B, E, F) for downstream qPCR and 16S rRNA gene amplicon analysis. The corresponding qPCR results are shown in panels C, D, G, and H. Panels B, F, D, and H show results from ATU-treated microcosms. Error bars represent standard deviations of biological replicates (*n* ≥ 3). Different letters denote statistically significant differences among treatments, including the initial time point. Statistical analyses were performed separately for each ammonia oxidizer group as described in the Methods section (*P* < .05).

To investigate the response of ammonia oxidizers to salinity changes in freshwater reservoirs, microcosms were prepared using unfiltered natural water samples from freshwater reservoirs Soyang and Daecheong (Supplementary Table S2), each retaining its intrinsic salinity. The microcosms containing supplements such as trace metals but no added basic salts (0× MWM; Supplementary Table S1) already exhibited ECs of ~170 μS/cm, representing lower salinity conditions of this study. In contrast, the addition of 10× MWM salts yielded ECs of ~870 μS/cm, representing higher salinity. In both freshwater microcosms, ammonia oxidation was completed within 10–25 days of incubation, and nitrite production rates (in these experiments, nitrite accumulated instead of nitrate) remained consistent across salinity levels (Fig. 3A and E). This nitrite accumulation may reflect rapid ammonia-oxidizing activity, with limited subsequent oxidation by nitrite-oxidizing bacteria. After ammonia oxidation was completed, AOB *amoA* gene abundance exceeded that of other ammonia oxidizers regardless of salt supplementation (Fig. 3C and G). This consistent dominance suggests that freshwater-adapted AOB had a competitive advantage relative to other ammonia oxidizers.

In the presence of ATU, ammonia oxidation was delayed, requiring ~90–180 days for completion (Fig. 3B and F). Similar to patterns observed in soil microcosms, nitrate production was faster at elevated salinity (10× MWM). To determine whether reservoir water itself suppresses the nitrification activity of AOA, *N. koreense* MY1 was inoculated into microcosms containing unfiltered water from the Soyang reservoir with ATU (Supplementary Fig. S3). Upon inoculation of the strain, ammonia oxidation under 10× MWM conditions was completed much faster than in the uninoculated microcosms (Fig. 3B), within 26 days (Supplementary Fig. S3). However, ammonia oxidation remained minimal and incomplete under 0× MWM conditions. This demonstrates that *N. koreense* MY1 was not inhibited by reservoir water itself, whereas the sensitivity of AOA to low salinity conditions remained evident in the microcosms. Moreover, the prolonged delay in archaeal ammonia oxidation under high salinity conditions in the presence of ATU is likely attributable to low initial abundance or low viability of AOA cells, or to a prolonged resuscitation time of dormant AOA cells in the original reservoir water. This is also consistent with these water reservoirs not being AOA-dominated systems, unlike oligotrophic lakes where AOA are the primary drivers of ammonia oxidation [28]. Although AOA *amoA* gene was predominant after ammonia oxidation regardless of salt supplementation (Fig. 3D and H), a slight increase in AOB and CMX-A/B *amoA* gene abundances was also observed under ATU treatment, similar to the soil microcosms (Fig. 1D and H), possibly reflecting incomplete inhibition or partial loss of ATU efficacy [85]. Together, these findings indicate that AOA in freshwater are subjected to stress under low salinity conditions, leading to reduced nitrification activity.

### Freshwater reservoir ammonia-oxidizing community analysis

**Figure 4:**
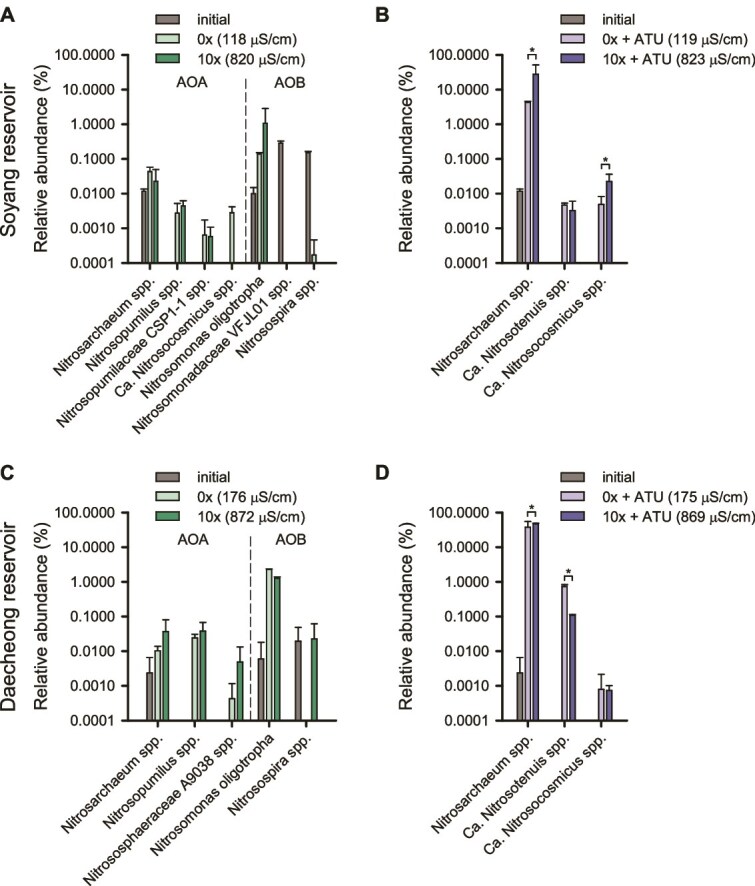
Relative abundances of ammonia oxidizers in freshwater microcosm samples. The relative abundances (as % of total 16S rRNA gene reads) of ammonia oxidizers at the genus level are shown for microcosms derived from Soyang reservoir (A, B) and Daecheong reservoir (C, D). Panels B and D show results from the ATU-treated microcosms. Taxa detected in only a single replicate across all treatments or with abundances below 0.002% were excluded from the analysis. Error bars represent standard deviations of biological replicates (*n* ≥ 3). Initial samples were excluded from statistical analyses. Asterisks indicate statistically significant differences among treatments based on DESeq2 analysis of raw count data (adjusted *P* ≤ .05). ASV-level information for all ammonia oxidizers detected, including AOB and *Nitrospira* in ATU-treated microcosms, is provided in Supplementary Dataset S1. Species-level differential abundance results based on DESeq2 are provided in Supplementary Dataset S4.

Unlike in the soil microcosms, there was no significant difference in the relative abundance of ammonia oxidizers between low- and high-salinity conditions in freshwater microcosms in the absence of ATU. AOB affiliated with the *N. oligotropha* lineage were predominant in the ammonia-oxidizing community after the completion of ammonia oxidation in both freshwater microcosms (Fig. 4A and C). The composition of ASVs within the *N. oligotropha* lineage was diverse across both freshwater reservoirs and salinity levels, with no single ASV dominating (Dataset S1), suggesting extensive microdiversity at the ASV level, potentially reflecting their adaptation to oligotrophic and low salinity environments [87]. In contrast to the soil microcosms, which were dominated by the genus “*Ca*. Nitrosotenuis,” the genera *Nitrosarchaeum* (ASV271 and ASV909) and *Nitrosopumilus* (ASV135 and ASV827) within AOA-NpF (Supplementary Fig. S2A; Dataset S1) were relatively more represented within the AOA community in freshwater microcosms (Fig. 4A and C), regardless of salinity. ASV271 and ASV909 were closely related (>98.9% identity) to *N. koreense* MY1, and ASV135 and ASV827 showed >99.5% identity to *Nitrosopumilus maritimus* SCM1 (Supplementary Fig. S2A; Dataset S1). In the presence of ATU, AOA in the genus *Nitrosarchaeum* (ASV271 in freshwater reservoir Soyang and ASV909 in freshwater reservoir Daecheong) became the dominant ammonia oxidizers (Figs 3B and F;  4B and D).

Taken together, results from the soil microcosms showed a significant increase in AOB abundance at lower salinity levels (Fig. 1C and G), possibly due to the inhibition of AOA or CMX-A (Fig. 1B, C, F, and G), thereby providing a competitive advantage to AOB. In contrast, AOA became the most dominant group after ammonia oxidation in soil microcosms under elevated salinity conditions (Fig. 1C and G). In the freshwater microcosms, AOB were predominant at both salinity levels, likely reflecting the lower initial abundance and less competitive response of AOA and CMX relative to AOB (Fig. 3). The dominant AOB detected in both soil and freshwater microcosms were affiliated with the *N. oligotropha* lineage (Figs 2A and C; 4A and C). In the freshwater microcosms, their predominance may partly reflect ecophysiological traits that confer competitive advantages over AOA and CMX under a transient increase in substrate availability following amendment with 25 μM NH_4_Cl. Under such conditions, AOB is often regarded as typical r-strategists compared with AOA [88, 89]. In addition, compared to other AOB, strains of the *N. oligotropha* lineage are known to exhibit a higher affinity for NH_3_ + NH_4_^+^ (*K*_m(app)_ = 4.4 μM) [20]. The dominant AOA detected in soil and freshwater microcosms were affiliated with the genera “*Ca*. Nitrosotenuis” and *Nitrosarchaeum*, respectively, which were selectively responsive to high salinity (Figs 2; 4B and D).

### Effects of hypoosmolarity on the growth of ammonia oxidizers

**Figure 5:**
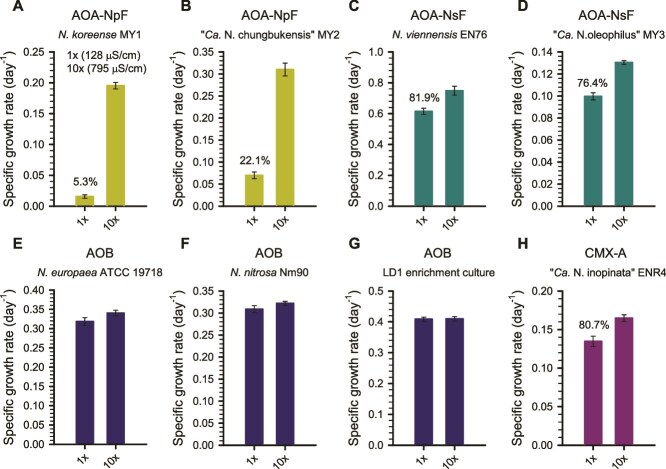
Growth of ammonia oxidizers under hypoosmotic conditions. Growth was compared between 1× and 10× MWM, representing the hypoosmotic and control conditions, respectively. The composition of MWM is provided in Supplementary Table S1. Error bars represent the standard deviation of biological replicates (*n* ≥ 3). For strains exhibiting significantly reduced growth under hypoosmotic conditions (two-tailed t-test*, P* < .05), the percentage decrease in growth rate relative to the control is shown.

The findings from soil and freshwater microcosm experiments indicated that the nitrification activity of ammonia oxidizers is differentially affected by low salinity within the range typical of natural environments (Supplementary Fig. S1). Thus, we hypothesized that chronic hypoosmolarity stress at low salinity levels affects AOA or CMX-A. To test this hypothesis, we examined the growth response of eight cultivated ammonia oxidizers—four AOA strains (AOA-NpF: *N. koreense* MY1 and “*Ca.* N. chungbukensis” MY2; AOA-NsF: *N. viennensis* EN76 and “*Ca.* N. oleophilus” MY3), two AOB cultures (*N. europaea* lineage: *N. europaea* ATCC 19718, *N. communis* lineage: *N. nitrosa* Nm90), one AOB enrichment culture (LD1) dominated by *N. oligotropha* lineage, and one CMX-A strain (“*Ca*. N. inopinata” ENR4)—across salinity levels from 0× to 128× MWM (71–2700 μS/cm), except for “*Ca*. N. oleophilus” MY3, which was tested only in 1× and 10× MWM (Fig. 5; Supplementary Fig. S4).

The growth rates of the three AOA strains were generally lower at 0–4× MWM (71–318 μS/cm) compared to 10× MWM, with statistically significant reductions observed for strains *N. koreense* MY1 and “*Ca.* N. chungbukensis” MY2, particularly at the lowest salinity levels (Fig. 5; Supplementary Fig. S4). Although *N. viennensis* EN76 also exhibited a significant reduction at 0× MWM, its growth rate declined less sharply across the salinity gradient, indicating a comparatively lower sensitivity to hypoosmolarity. At 10× MWM (795 μS/cm), the growth rates of “*Ca.* N. chungbukensis” MY2 and *N. viennensis* EN76 were comparable to those observed in AFM (3540 μS/cm), a conventional medium for cultivating ammonia oxidizers (Fig. 5; Supplementary Fig. S4) [54]. In contrast, the growth rates of all AOB cultures (*N. europaea* ATCC 19718, the LD1 enrichment culture, *N. nitrosa* Nm90) did not differ significantly across a variable salinity range (Fig. 5; Supplementary Fig. S4); the specific activity of AOB in the LD1 culture was verified (Supplementary Fig. S5). This supports observations from soil and freshwater microcosms in which AOB remained active under low-salinity conditions (63–176 μS/cm) (Figs 1–4). Likewise, the growth of the CMX-A strain, “*Ca*. N. inopinata” ENR4, differed between salinity conditions (Fig. 5; Supplementary Fig. S4), with slightly reduced growth observed under low-salinity conditions, consistent with the inhibition of CMX-A activity in low-salinity soil microcosms (Fig. 1C and G). “*Ca.* N. inopinata” ENR4 was isolated from a deep oil exploration well [3], which may account for physiological differences between this CMX-A and those CMX-A-members in moderate soil and freshwater environments. In fact, the dominant *Nitrospira* lineage II ASVs (ASV1054, ASV10596, ASV1971, ASV2125) detected in our microcosms were different from “*Ca.* N. inopinata” ENR4 (Supplementary Fig. S2C; Dataset S1).

Based on these findings, 1× MWM (128 μS/cm) and 10× MWM (795 μS/cm) were selected as hypoosmotic and control conditions for further analysis, respectively. At 1× MWM, AOA-NpF strains showed marked inhibition, with *N. koreense* MY1 and “*Ca*. N. chungbukensis” MY2 retaining only 7.9% and 22.5% of their growth rates relative to 10× MWM. AOA-NsF strains showed milder inhibition, with *N. viennensis* EN76 and “*Ca.* N. oleophilus” MY3 retaining 81.9% and 76.4%, respectively. In contrast, all AOB cultures showed no substantial change, and the CMX-A strain, “*Ca*. N. inopinata” ENR4, retained 80.7% of its growth rate relative to 10× MWM. These differences in inhibition across ammonia oxidizers were consistent with microcosm results, where AOA-NpF exhibited more pronounced low salinity-dependent inhibition (Figs 1–4). This pattern is further supported by slower growth rates of dominant AOA-NpF under lower salinity with ATU, as observed in both soil and freshwater microcosms (Figs 1–4). Overall, our results clearly indicate that among the ammonia oxidizers, AOA, particularly AOA-NpF, are especially sensitive to hypoosmolarity stress.

### Comparative genomic and gene expression analyses

Two key cellular properties may determine the sensitivity of microorganisms to hypoosmotic stress: (i) osmoregulatory systems that maintain ionic and osmotic balance [48, 49] and (ii) cell surface structures that provide resistance to turgor pressure. Hypoosmotic shock imperatively leads to an influx of water across the cell membrane, increasing cell volume and turgor pressure. In the absence of a rigid cell wall, this pressure ultimately results in membrane rupture and loss of structural integrity [50]. To elucidate the mechanisms underlying the adaptation of ammonia oxidizers to hypoosmolarity, we compared the genomic repertoires of osmoregulatory systems across AOA-NpF, AOA-NsF, AOB, and CMX (Supplementary Fig. S6; Dataset S2) and performed transcriptomic and proteomic analyses. Compared to AOA, AOB and CMX genomes contain various sensing and transport systems, primarily functioning in K^+^ or Na^+^ transport [51], which may provide greater flexibility in osmoregulation under hypoosmotic conditions (see Supplementary Text for details).

To reveal the mechanisms underlying sensitivity to hypoosmotic stress, transcriptomic and proteomic responses of *N. viennensis* EN76 and “*Ca.* N. chungbukensis” MY2 cells grown at 1× and 10× MWM were comparatively analyzed, revealing distinct transcriptional responses under hypoosmotic conditions (Supplementary Datasets S5–S9). In contrast, transcriptomic analysis of the AOB strain *N. europaea* ATCC 19718 revealed no detectable differences between 1× and 10× MWM (Supplementary Dataset S10), consistent with physiological results indicating negligible inhibition under hypoosmotic conditions.

**Figure 6:**
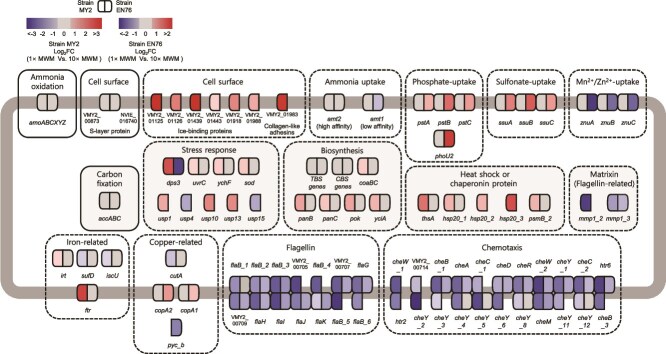
Differential gene expression under varying salinity conditions. A schematic overview of differentially expressed genes in “*Ca*. Nitrosotenuis chungbukensis” MY2 and *Nitrososphaera viennensis* EN76 under 1× MWM (128 μS/cm) vs. 10× MWM (795 μS/cm). Solid-bordered boxes denote constitutively expressed genes; dashed-bordered boxes represent differentially expressed genes grouped by function. The color scale indicates higher gene expression in cells grown under 1× MWM (red) or 10× MWM (blue). The intensity of each color reflects the magnitude of expression change (log_2_fold change). Genes with |fold change| < 1.5 are shown in gray. Genes involved in ammonia oxidation (*amoABCXYZ*), carbon fixation (*accABC*), tetrapyrrole biosynthesis (TBS genes; listed under the tetrapyrrole biosynthesis function category in Supplementary Dataset S9), and cobalamin biosynthesis (CBS genes; listed under the cobalamin biosynthesis function category in Supplementary Dataset S9) were each grouped and represented as a single box.

The depth of the proteome dataset for the AOA was limited, with 383 of 3114 predicted CDSs detected for EN76 (12.3%) and 635 of 2019 CDSs detected for MY2 (31.5%), which constrained the extent of downstream functional interpretation. Out of 3114 genes recovered from the transcriptome analysis of *N. viennensis* EN76, only 26 and 121 genes were significantly up-regulated and down-regulated at 1× MWM, respectively, based on a 1.5-fold change with an adjusted *P* < .01 (Supplementary Datasets S5 and S7). In the case of “*Ca.* N. chungbukensis” MY2, 139 and 168 genes were significantly up-regulated and down-regulated at 1× MWM, respectively, out of 2019 genes recovered from the transcriptome analysis (Supplementary Datasets S6 and S8). Most central metabolism genes involved in ammonia oxidation, electron transport chain, CO_2_ fixation, TCA Cycle, and pentose phosphate pathway were constitutively expressed in both strains. However, genes differentially expressed under hypoosmotic conditions (1× MWM) belong to transport, cell surface, stress response, protein synthesis, proteolysis, biosynthesis, motility, and chemotaxis (Fig. 6; Supplementary Datasets S5–S9).

#### *Transporters* 

In *N. viennensis* EN76, genes encoding nutrient transporters were the most differentially expressed dependent on salinity. The nutrient transporter genes upregulated under 1× MWM were related to the transport of copper (*cop*), phosphate (*pst*), and aliphatic sulfonate (*ssu*) (Fig. 6; Supplementary Dataset S9). Conversely, genes for low-affinity ammonium transporter (*amt2*) and ABC-type Mn^2+^/Zn^2+^ transport system (*znu*) were downregulated under 1× MWM. In the case of “*Ca.* N. chungbukensis” MY2, high-affinity Fe^2+^/Pb^2+^ permease (*ftr*) was upregulated under 1× MWM (Fig. 6; Supplementary Dataset S9).

Although the rationale for the upregulation of genes involved in ionic nutrient and metal uptake under hypoosmolarity conditions remains unclear, we suspect that this may be due to ionic gradient disruption across the membrane [90–92] or moderately low cation bioavailability at low salinity. Contrary to expectations, the genes for many other osmoregulatory systems, including Trk and Kdp potassium transporters, mechanosensitive channels, and aquaporin analyzed above (Supplementary Text; Fig. S6; Dataset S2), were constitutively expressed in both AOA strains (Supplementary Dataset S9). Together, these transcriptional responses suggest that low salinity alters the expression of genes related to nutrient uptake more prominently than those involved in osmoregulatory systems.

#### *Cell surface structure* 

The sensitivity of AOA to hypoosmolarity may also be attributed to archaeal-specific features such as their cell wall and envelope structures. In contrast to bacterial cells, which have semirigid cell walls and a high turgor pressure [93–95] due to peptidoglycan layers, archaea contain an S-layer as their main cell envelope structure, which may compromise AOA cells’ structural resilience under low salinity conditions. For example, when haloarchaeal cells are exposed to dilute environments, they lose the integrity of their S-layer lattice due to lack of divalent cations [96, 97]. Also, AOA cells are vulnerable to physical stress during centrifugation and filtration, whereas AOB cells are not [68, 98–100]. Comparatively, the number of genes related to membrane biosynthesis and cell envelope functionality is significantly higher in AOA-NsF than in AOA-NpF [101]. Further, cells in AOA-NsF are coccoid, in contrast to the rod-shaped cells of AOA-NpF. *Nitrosopumilus maritimus* SCM1 (AOA-NpF) has a hexagonal (p6) symmetry S-layer lattice [102], whereas *N. viennensis* EN76 (AOA-NsF) has a p3 symmetry S-layer lattice [69]. Together, possible differences in osmoregulatory systems and cell wall structures may explain the higher sensitivity of AOA, especially AOA-NpF clade, to hypoosmolarity. However, genes encoding the S-layer were not differentially expressed in both AOA strains (Fig. 6; Supplementary Dataset S9). Although both AOA lineages possess an S-layer, *N. viennensis* was also able to tolerate low salinity better than “*Ca.* N. chungbukensis” MY2. Thus, S-layer alone cannot account for the markedly different salinity tolerances observed between AOA and AOB, indicating that additional structural and physiological factors may also contribute to their divergent responses.

In contrast to *N. viennensis* EN76, which lacked differentially expressed genes related to cell surface proteins, most of the genes upregulated in “*Ca.* N. chungbukensis” MY2 cells grown under 1× MWM were associated with cell surface structures (Fig. 6; Supplementary Dataset S9). Genes encoding ice-binding proteins (IBPs; also known as antifreeze proteins), LPXTG-anchored collagen-like adhesins were highly expressed under this condition. Especially, five of the eight IBP genes ranked within the top 100 most highly expressed transcripts in 1× MWM, and their corresponding proteins alongside a LPXTG-anchored collagen-like adhesin were also detected in the proteome of 1× MWM-grown cells (Supplementary Dataset S6). As cells of “*Ca.* N. chungbukensis” were grown at 30°C, the expression of IBP-like genes was unexpected. Four of the five IBP contain additional C-terminal domains of unknown function (Supplementary Fig. S7), which may indicate a broader role of these proteins, including “*Ca*. N. chungbukensis” MY2 cell envelope stabilization in dilute environments. IBP are additionally suggested to be involved in protecting membrane stability [103–105]. Furthermore, LPXTG-anchored collagen-like adhesin may interact with the S-layer to form macromolecular surface assemblies [106, 107], which could serve as a protective mechanism to prevent structural damage in AOA cells under hypoosmotic stress. Therefore, their increased expression under low-salinity conditions may represent a physiological response to maintain cell envelope stability.

#### *Others* 

Both strains downregulated the expression of motility-associated genes, including those encoding flagellin and chemotaxis-related proteins, which are generally clustered in the genome, under 1× MWM (14 and 22 genes, respectively) (Fig. 6; Supplementary Dataset S9). This downregulation has been widely reported under various stress conditions and is often interpreted as a metabolic shift that prioritizes stress tolerance over motility [108–110]. Differentially expressed genes involved in stress response and signal transduction, protein synthesis and degradation, and biosynthesis are described in the Supplementary Text. These suggest that low-salinity stress may trigger a broader physiological adjustment affecting cellular homeostasis.

Together, our findings demonstrate that AOA cells differ fundamentally from AOB cells in osmoprotection strategies due to differences in: (i) the osmoregulatory transport system and (ii) cell surface structure [93–98]. These differences likely contribute to the heightened sensitivity of AOA, especially AOA-NpF, to hypoosmolarity stress, as observed in this study. Consistent with this, the AOB *N. europaea* ATCC 19718 exhibited no detectable transcriptional changes under hypoosmotic conditions (Supplementary Dataset S10), indicating effective osmotic buffering without transcriptional reprogramming. In addition, representative isolates from two AOA clades used in this study exhibited distinct transcriptional responses to hypoosmotic stress, reflecting their different degrees of sensitivity. *N. viennensis* EN76 exhibited limited transcriptional changes (mainly related to nutrient uptake) (Supplementary Dataset S9), whereas “*Ca.* N. chungbukensis” MY2, closely related to the highly responsive AOA-NpF abundant in higher salinity soil and freshwater microcosms, showed broad transcriptional changes (cell surface and envelope-associated proteins, stress response and signal transduction, and chemotaxis) (Supplementary Dataset S9), indicative of severe stress under hypoosmotic conditions. Our results indicate that osmoprotection under hypoosmolarity is closely linked to cell wall rigidity, and the lack of a peptidoglycan-like cell wall in AOA likely plays an important role in their heightened sensitivity to this stress.

### Ecological relevance of hypoosmolarity

Salinity is a key determinant of microbial community structure in various terrestrial and freshwater environments [9]. A freshwater microcosm study using a forested watershed stream showed that microbial diversity declined at lower salinity levels (<350 μS/cm) [36]. In the same study, the relative abundance of the phylum *Nitrososphaerota* (National Center for Biotechnology Information classification system), harboring AOA, began to increase only when the conductivity exceeded 350 μS/cm [36], highlighting AOA sensitivity to low salinity. The upper bound of this range is comparable to the biological effect concentration of 300 μS/cm (HC_05_; the fifth percentile hazardous concentration) reported for other aquatic organisms [37, 38], suggesting a potential biological threshold for hypoosmolarity at which many organisms, including AOA, may experience osmotic stress.

Consistent with these environmental observations, our microcosm and physiological studies strongly support that AOA, especially members of AOA-NpF, experienced growth inhibition under hypoosmotic conditions (<300 μS/cm), whereas AOB were largely unaffected (Figs 1–5; Supplementary Fig. S4). In particular, the genus *Nitrosarchaeum* (members of AOA-NpF), exhibited significantly delayed growth under hypoosmotic conditions in microcosms from the freshwater reservoirs Soyang and Daecheong (Figs 3 and 4). These water reservoirs, like many others in Korea, are exorheic with relatively short water residence times (<0.75 years) and receive continuous freshwater inflow, resulting in persistently hypoosmotic conditions (<180 μS/cm) [111–113]. Given the heightened sensitivity of AOA-NpF to low salinity, together with the rapid flushing of these systems, their long-term persistence is likely constrained. In Eurasian lakes with long water residence times and persistent hypolimnetic layers, “*Ca.* Nitrosopumilus limneticus” of AOA clades dominates other ammonia oxidizers even under hypoosmotic conditions [28]. Prolonged hydrological retention could allow AOA to maintain dominance despite limited in situ population renewal [114]. Thus, the scarcity of AOA in our studied freshwater reservoirs likely reflects the combined effects of persistent hypoosmolarity and short residence times, which together limit opportunities for their accumulation.

In contrast, AOB, which are more resistant to hypoosmolarity, may be favored under these conditions, as reflected in their higher activity in both microcosm and physiological studies (Figs 1, 3, and 5). This pattern is consistent with observations from other freshwater systems [27, 29, 30], where AOB and AOA co-occurred and AOB often outnumbered AOA even in environments where ammonium concentrations would otherwise have favored AOA growth [27, 29, 30]. In Flathead Lake (150–172 μS/cm [115]) and the Laurentian Great Lakes (79–334 μS/cm [116–120]), maximum NH_4_^+^ concentrations reach ~0.5 μM with marked seasonal variability [29, 30, 121, 122]. It can be assumed that lake-associated AOB (*Nitrosospira*) possess higher affinities to ammonia than the values (*K*_m(app)_ = ~150 μM [123]) inferred from cultured representatives considering a clear increase of AOB in increased summer ammonia up to 0.5 μM in the water column [30]. A cultivated strain of *Nitrosarchaeum* has a higher affinity for NH_3_ + NH_4_^+^ (*K*_m(app)_ = 0.56 μM [20]). In high-altitude lakes of the Sierra Nevada, where conductivities range from 1.8 to 94.2 μS/cm [124] and NH_4_^+^ concentrations range between 2.8 and 72 nM, AOB consistently outnumbered AOA [27]. In the Laurentian Great Lakes, it was also suggested that AOB survival may be enhanced by the acquisition of proteorhodopsin genes in the photic layers [29]. Thus, additional niche-determining factors beyond ammonium availability may contribute to the observed patterns.

In soil environments, salinity is highly dynamic because it fluctuates with wetting–drying cycles caused by rainfall, evaporation, and irrigation [125], and is therefore difficult to define due to spatiotemporal heterogeneity and fluctuating moisture regimes [126]. Further, most previous studies on the effects of salinity on microbial processes including nitrification have focused on hyperosmolarity, as high salinity is often a serious concern in agricultural soils [8, 10, 31–35]. In Antarctic Dry Valley soils, higher AOA/AOB ratios were associated with higher EC, suggesting that AOB may be more competitive under low EC conditions [127]. Consequently, empirical evidence directly linking low salinity to ammonia oxidizer dynamics in soils remains limited, highlighting the need for more systematic investigation of how low salinity shapes soil ammonia oxidizer communities.

## Conclusions

Although EC in terrestrial and freshwater environments is much lower than in general microbiological media, the responses of ammonia oxidizers to chronic hypoosmotic stress remain poorly understood. In this study, we show that AOA, particularly members of AOA-NpF, are highly sensitive to low-salinity conditions commonly encountered in soil and freshwater environments. In contrast, AOB displayed greater tolerance to low salinities and frequently dominated under such conditions. Genomic and transcriptomic analyses suggest that members of AOA-NpF possess limited osmoprotective capacity, possibly due to the lack of (i) robust osmoregulatory systems and (ii) of a rigid cell surface structure. In hydrological settings where increased precipitation or freshwater inflow is expected to lower salinity, understanding the impact of chronic hypoosmotic stress on terrestrial and freshwater nitrogen cycling can be important. Hypoosmolarity-induced shifts in ammonia-oxidizing community composition could have important implications for nitrogen management practices and strategies to mitigate N_2_O emissions in those terrestrial and freshwater environments. This study highlights osmotic stress as a potentially underrecognized but ecologically relevant factor influencing ammonia-oxidizing community dynamics in low-salinity environments.

## Supplementary Material

wrag045_Hypoosmolarity_SI

wrag045_Hypoosmolality_SI_Datasets

## Data Availability

The transcriptomic and 16S rRNA gene amplicon sequencing data have been deposited in the NCBI BioProject under the accession number PRJNA1265630. The mass spectrometry-based proteomics data have been deposited to the ProteomeXchange Consortium via the PRIDE [128] partner repository under the dataset identifier PXD064500.
