# Supplementary material for: Hypoosmolarity inhibits ammonia oxidation by terrestrial and freshwater Nitrosopumilaceae members
Source: ISME J. 2026 Mar 6;20(1):wrag045. doi: 10.1093/ismejo/wrag045 (PMC13037477; doi:10.1093/ismejo/wrag045)
Supplement: wrag045_Hypoosmolarity_SI [file wrag045_hypoosmolarity_si.docx]

**Hypoosmolarity inhibits ammonia oxidation by terrestrial and freshwater *Nitrosopumilaceae* members**

# Short title: Hypoosmolarity in nitrification

Joo-Han Gwak^1,2,‡^, Adebisi Olabisi^3,‡^, Ui-Ju Lee^3^, Christiana Abiola^3^, Seongjun Lee^3^, Hackwon Do^4^, Yun Ji Choi^5^, Jay-Jung Lee^6^, Man-Young Jung^5,7^, Nico Jehmlich^8^, Martin von Bergen^8,9^, Michael Wagner^10,11^, Samuel Imisi Awala^12^, Zhe-Xue Quan^13^, and Sung-Keun Rhee^3,*^

^1^Department of Life Science, Hallym University, Chuncheon 24252, Republic of Korea
^2^Multidisciplinary Genome Institute, Hallym University, Chuncheon 24252, Republic of Korea
^3^Department of Biological Sciences and Biotechnology, Chungbuk National University, 28644 Cheongju, Republic of Korea ^4^Division of Life Sciences, Korea Polar Research Institute, 21990 Incheon, Republic of Korea
^5^Interdisciplinary Graduate Program in Advanced Convergence Technology and Science, Jeju National University, 63243 Jeju, Republic of Korea
^6^Geum River Environment Research Center, National Institute of Environmental Research, 29027 Okcheon, Republic of Korea
^7^Department of Biology Education, Jeju National University, 63243 Jeju, Republic of Korea
^8^Department of Molecular Systems Biology, Helmholtz Centre for Environmental Research–Zentrum für Umweltforschung GmbH, 04318 Leipzig, Germany
^9^Institute of Biochemistry, Faculty of Biosciences, Pharmacy and Psychology, University of Leipzig, 04103 Leipzig, Germany
^10^Centre for Microbiology and Environmental Systems Science, Department of Microbiology and Ecosystem Science, University of Vienna, Vienna, Austria
^11^Department of Chemistry and Bioscience, Aalborg University, Aalborg, Denmark
^12^Department of Biological Sciences, University of Calgary, Calgary, AB T2N 1N4, Canada
^13^School of Life Sciences, Fudan University, Shanghai, China

^‡^J-HG and AO contributed equally to this work.

*Corresponding author.
**Email:** [rhees@chungbuk.ac.kr](mailto:rhees@chungbuk.ac.kr) **Phone**: +82-43-261-2300. Fax: 82-43-264-9600.

**Keywords:** Hypoosmolarity, Nitrification, Soil and freshwater ecosystems, Ammonia-oxidizing archaea

**This PDF file includes:**

Supplementary Text

Supplementary Tables S1 to S3

Supplementary Figures S1 to S7

Legends for Supplementary Datasets S1 to S10

Supplementary References

**Other supplementary materials for this manuscript include the following:**

Datasets S1 to S10

# Supplementary Text

## *Comparative genomic analysis of osmoregulation systems in ammonia oxidizers*

**K^+^ transport systems:** A distinct difference in K^+^ transport systems was observed between AOB and other ammonia oxidizers (AOA and CMX). Genes encoding K^+^ transport proteins — such as putative flavoprotein involved in K^+^ transport, K^+^ uptake Trk/Ktr system TrkAH (TC 2.A.38.1, 2.A.38.4), multicomponent K^+^:H^+^ antiporter Pha (TC 2.A.63.1), and K^+^:H^+^ antiporter Kef (TC 2.A.37.1) — were found exclusively in AOB (Supplementary Dataset S1). Most bacterial ammonia oxidizers (AOB and CMX), in contrast to AOA, also encoded Na^+^:2H^+^ antiporter NhaA (TC 2.A.33) or Na^+^:H^+^ antiporter NhaC (TC 2.A.35), and Mg^2+^ transporter MgtE (TC 1.A.26.1.6) (Supplementary Fig. S6; Dataset S2). These systems primarily function in K^+^ or Na^+^ efflux and may provide greater flexibility in osmoregulation under hypoosmotic conditions [1], particularly in AOB and CMX.

The Trk system typically operates as a complex of TrkA and TrkH, where TrkA serves as a regulatory subunit [2]. In AOA, however, only *trkH* homologs were detected (Supplementary Fig. S6; Dataset S2). In the absence of TrkA, TrkH is likely to function as a non-selective cation channel permeable to K^+^ or Na^+^ [2, 3]. This configuration may lead to uncontrolled K^+^ flux, which could impair homeostatic control under hypoosmotic conditions. By contrast, the high-affinity K^+^ transporter KdpABC (TC 3.A.3.7; *K*_m_ = ~2 μM [4]) was identified in several genomes of AOA-NsF, AOB, and CMX (Supplementary Fig. S6; Dataset S2), including strains that exhibited little or no growth inhibition under low-salinity conditions (Fig. 5). The presence of this high-affinity K^+^ uptake system may provide a selective advantage in mitigating hypoosmotic stress.

The low-salinity-insensitive strain *N. europaea* ATCC 19718 (Fig. 5) encodes only the TrkAH system for K^+^ uptake, along with two K^+^:H^+^ antiporters (YbaL and Pha), a voltage-gated K^+^ channel, and the mechanosensitive channel MscK, which requires external K^+^ for activation (Supplementary Fig. S6; Dataset S2). K^+^ transport in *N. europaea* is electrogenically driven [5]. Notably, even after potassium depletion via diethanolamine treatment, respiration with NH_4_^+^ retained 83% of the untreated activity, and the proton-motive force was preserved in the absence of added K^+^ [5]. These observations may indicate that strain *N. europaea* ATCC 19718 can sustain osmotic and energetic homeostasis with relatively low sensitivity to external K^+^ availability. The two K^+^:H^+^ antiporters may further support low-K^+^ adaptation by facilitating proton-coupled K^+^ uptake [5], particularly at concentrations above 50 μM (1× MWM; Supplementary Table S1; Fig. S6).

**Aquaporins and mechanosensitive channels:** The genomic survey revealed distinct patterns in water-channel (aquaporin) genes and mechanosensitive channel genes across ammonia oxidizers. Cells can regulate the activity of aquaporins to rapidly adjust the permeability of membranes to water [6-8]. Most AOA-NpF genomes encoded two aquaporin types, whereas AOA-NsF harbored only one and aquaporins were largely absent from AOB and CMX (Supplementary Fig. S6; Dataset S2). AOA-NsF, AOB, and CMX exhibited greater tolerance under low-salinity conditions (Fig. 5). This absence of aquaporins in AOB and CMX suggests a lineage-specific selective loss (aquaporins are not universally retained in prokaryotes [9]), although the physiological advantage of dispensing with dedicated water channels under natural hypoosmotic conditions remains uncertain.

Genes encoding low-threshold MscS/MscM mechanosensitive channels were broadly present across AOA-NsF, the majority of AOB, and CMX genomes but were absent from most of AOA-NpF (Supplementary Fig. S6; Dataset S2). These channels are small-conductance pores that open at modest membrane tension, serving as early-response “safety valves” under sudden hypoosmotic stress by slowly releasing osmolytes and relieving intracellular pressure [10, 11]. In contrast, the large-conductance mechanosensitive channel MscL was found in a subset of AOA-NpF and AOA-NsF genomes, as well as across CMX genomes, but was absent in AOB genomes (Supplementary Fig. S6; Dataset S2). MscL is activated at high membrane tension during osmotic down-shock, forming large non-selective pores that rapidly release cytoplasmic osmolytes [12]. However, experimental studies demonstrate that MscL alone does not ensure proper termination of the osmotic permeability response and instead flickers near its gating threshold, causing prolonged leakage when low-threshold channels are absent [10, 11, 13]. Most of AOA-NpF encode an intermediate-conductance mechanosensitive channel MscK, whose activation requires both membrane tension and extracellular K^+^ ions [14], while lacking low-threshold MscS/MscM channels (Supplementary Fig. S6; Dataset S2). The dual gating mechanism suggests that MscK-mediated pressure buffering is contingent on environmental ionic composition. In freshwater or persistently hypoosmotic habitats characterized by low external K^+^ concentrations, MscK may be inefficiently activated despite mechanical stress, functionally limiting its role as a safety valve. Members of *N. oligotropha* lineage also encode only MscK; however, AOB possess diverse K^+^ transport systems described above (Supplementary Fig. S6; Dataset S2), which may partially compensate for limited mechanosensitive buffering capacity. In contrast to other ammonia oxidizers, the AOA lineage “*Ca.* Nitrosopumilus limneticus”, which dominates in hypoosmotic lake environments, completely lacks detectable mechanosensitive channels across its genome (Supplementary Fig. S6; Dataset S2). In relatively stable, chronically diluted lake environments, this absence may reduce unnecessary cytoplasmic solute loss and prevent recurrent energetic investment in compatible solute replenishment.

These genomic differences may also reflect broader ecological trends, as phylogenomic analyses suggest that AOA-NpF derive from marine ancestors [15] that experienced relatively stable, higher-salinity regimes, whereas AOA-NsF evolved in soil environments characterized by frequent osmotic fluctuations. Thus, the presence of MscS/MscM in AOA-NsF, AOB, and CMX (Supplementary Fig. S6; Dataset S2) may confer a physiological advantage under hypoosmotic conditions by buffering osmotic shock through coordinated low-threshold mechanosensitive channels and associated ion transport systems, compared with AOA-NpF that rely primarily on MscK.

## *Transcriptomic and proteomic responses of AOA to hypoosmolarity*

**Stress response and signal transduction:** The transcriptomes of “*Ca.* N. chungbukensis” MY2 displayed a significant number of upregulated genes involved in stress response and signal transduction in 1× MWM in contrast to those of *N. viennensis* EN76 (Fig. 6; Supplementary Dataset S9). Five genes of universal stress protein, four genes of heat shock protein, *uvrC* (nucleotide excision repair gene), *dps* (DNA protection against starvation, superoxide dismutase, and Fe^2+^-trafficking protein for iron homeostasis), and *ychF* (redox-regulated ATPase) were upregulated under 1× MWM. Several signal transduction protein genes were differentially expressed, showing both up- and downregulation.

**Protein synthesis and degradation**: In *N. viennensis* EN76, a gene for trypsin-like peptidase domain-containing protein was upregulated under 1× MWM. “*Ca*. N. chungbukensis” MY2 showed upregulation of genes functioning as chaperone, translocase, and elongation factor (Supplementary Dataset S9).

**Amino acid and cofactor biosynthesis**: Upregulation of genes involved in amino acids and NAD biosynthesis, such as 2-isopropylmalate synthase and quinolinate synthase, was observed under the 1× MWM in *N. viennensis* EN76. In contrast, many genes related to nucleotide, lipid, sugar, and vitamin (coenzyme) metabolism were upregulated in “*Ca*. N. chungbukensis” MY2 (Fig. 6; Supplementary Dataset S9).

# Supplementary Tables

## Table S1. Composition of the mineral water medium (MWM)^a^ and artificial freshwater medium (AFM) used in this study.

| Medium | 0× | 0.5× | 1× | 2× | 4× | 8× | 10× | 16× | 32× | 64× | 128× | AFM |
| --- | --- | --- | --- | --- | --- | --- | --- | --- | --- | --- | --- | --- |
| Conductivity^b^ (μS/cm) | 71 | 96 | 128 | 188 | 318 | 629 | 795 | 900 | 1,169 | 1,707 | 2,700 | 3,540 |
| Basic salts |  |  |  |  |  |  |  |  |  |  |  |  |
| NaCl (mM) | - | - | - | 0 | 0 | 0.7 | 0.9 | 1.5 | 3.1 | 6.3 | 12.7 | 17.1 |
| KCl (mM) | - | - | - | 0.05 | 0.15 | 0.35 | 0.45 | 0.75 | 1.55 | 3.15 | 6.35 | 6.71 |
| MgSO_4_·7H_2_O (mM) | - | 0.022 | 0.045 | 0.09 | 0.19 | 0.4 | 0.5 | 0.5 | 0.5 | 0.5 | 0.5 | 1.97 |
| CaCl_2_·2H_2_O (mM) | - | 0.09 | 0.18 | 0.38 | 0.78 | 1.6 | 2 | 2 | 2 | 2 | 2 | 0.68 |
| Supplementary amendments |  |  |  |  |  |  |  |  |  |  |  |  |
| NH_4_Cl (mM)^c^ | 0.025 or 0.1 (for microcosms); 0.1 or 0.5 (for isolates and LD1) | | | | | | | | | | | |
| KH_2_PO_4_ (mM) | 0.05 (for microcosms, isolates, and LD1); 1.47 (for AFM) | | | | | | | | | | | |
| NaHCO_3_ (mM) | 0.1 (for microcosms, isolates, and LD1); 2 (for AFM) | | | | | | | | | | | |
| HEPES (mM)^c^ | 0.5 or 1 (for isolates and LD1); 2 (for microcosms) | | | | | | | | | | | |
| Pyruvic acid (mM) | 0.1 (for MY1, MY2, EN76); not added to other strains | | | | | | | | | | | |
| Trace elements^d^ (×) | 1 | | | | | | | | | | | |
| Ferric sodium EDTA (μM) | 7.5 | | | | | | | | | | | |
| Allylthiourea ATU (μM) | 50 (for microcosms) | | | | | | | | | | | |
| Total metal ions at pH 7.0 |  |  |  |  |  |  |  |  |  |  |  |  |
| Na^+^ (mM) | 0.35 | 0.6 | 0.6 | 0.6 | 0.6 | 1.3 | 1.5 | 2.1 | 3.4 | 6.9 | 10.4 | 21 |
| K^+^ (mM) | 0.01 | 0.01 | 0.05 | 0.1 | 0.2 | 0.4 | 0.5 | 0.8 | 1.36 | 3.2 | 4.51 | 8.18 |
| Mg^2+^ (mM) | 0 | 0.022 | 0.045 | 0.95 | 0.19 | 0.4 | 0.5 | 0.5 | 0.5 | 0.5 | 0.5 | 1.97 |
| Ca^2+^ (mM) | 0 | 0.09 | 0.18 | 0.38 | 0.78 | 1.6 | 2 | 2 | 2 | 2 | 2 | 0.68 |

^a^For the soil slurry microcosms and cultivation of isolates or enrichment culture, MWM was prepared using distilled water. For the freshwater microcosms, MWM was prepared directly using freshwater water.

^b^Conductivity values correspond to media adjusted to pH 7.0 with 0.1 mM NH_4_Cl, 0.05 mM KH_2_PO_4_, 0.1 mM NaHCO_3_, and 1 mM HEPES.

^c^For *Nitrosomonas europaea* ATCC 19718, *Nitrosomonas nitrosa* Nm90, and “*Candidatus* Nitrospira inopinata” ENR4, 0.5 mM NH_4_Cl was used; in contrast, 0.1 mM NH_4_Cl was used for all other strains or the LD1 enrichment culture.

^d^For experiments using 0.5 mM NH_4_Cl, 0.5 mM HEPES was used, whereas for experiments using 0.1 mM NH_4_Cl, 1 mM HEPES were used.

^e^Trace elements were prepared as described by Widdel and Bak [16].

## Table S2. Description of the location and properties of soil and freshwater samples for the microcosm.

| Sample | Grassland soil | Agricultural soil | Soyang  reservoir | Daecheong reservoir |
| --- | --- | --- | --- | --- |
| Location (GPS) | 36°37‘36.81“ N,  127°27‘20.31“ E | 36°37‘26.76“ N,  127°27‘14.40“ E | 37°56‘56.8“ N,  127°49‘04.3“ E | 36°28‘26.40“ N,  127°29‘13.56“ E |
| Sampling date | 2023-11 | 2023-11 | 2024-03 | 2024-05 |
| Sampling depth (m) | 0.5 | 0.5 | 20 | 20 |
| Conductivity (μS/cm) | 24.6 | 120.9 | 81.2 | 147.5 |
| Water content (%) | 25.1 | 36.8 | – | – |
| Residence time (yr) |  |  | 0.75 | 0.5 |
| Mixing regime |  |  | Monomictic | Monomictic |
| Trophic state^a^ |  |  | Oligotrophic | Mesotrophic |
| Average volume (km^3^) |  |  | 2.9 | 0.79 |
| Maximum depth (m) |  |  | 118 | 50 |
| Temperature (°C) | – | – | 6 | 15 |
| DO (mg/L) |  |  | 10.1 | 9.2 |
| Chlorophyll (µg/L) |  |  | 0.8 | 1.3 |
| pH | 5.78 | 7.94 | 6.98 | 6.70 |
| PO_4_^3−^(mg·N·kg^−1^ soil or μM) | n.d. | n.d. | <1 uM | <1 uM |
| NO_2_^−^(mg·N·kg^−1^ soil or μM) | n.d. | n.d. | n.d. | n.d. |
| NO_3_^−^ (mg·N·kg^−1^ soil or μM) | 9.8 | 13.6 | 94.2 | 91.9 |
| NH_4_^+^ (mg·N·kg^−1^ soil or μM) | 3.2 | 9.6 | 5.7 | 7.1 |

^a^Trophic state classification follows long-term monitoring and trophic state index-based assessments from previous studies [17, 18].

## Table S3. Primers used for qPCR quantification of AOA, AOB, and CMX *amoA* genes.

| Target | Primer name | Sequence | Condition | Reference |
| --- | --- | --- | --- | --- |
| AOB *amoA* gene | amoA-1F | GGGGTTTCTACTGGTGGT | 95°C for 5 min; 40 cycles of 95°C for 30 s, 60°C for 30 s, 72°C for 30 s; 72°C for 5 min |  |
|  | amoA-2R | CCCCTCKGSAAAGCCTTCTTC |  |  |
| AOA amoA gene | CamoA-19f | ATGGTCTGGYTWAGACG | 95°C for 5 min; 40 cycles of 95°C for 30 s, 60°C for 30 s, 72°C for 30 s; 72°C for 5 min |  |
|  | CamoA-616r | GCCATCCABCKRTANGTCCA |  |  |
| CMX clade A *amoA* gene | CA377f | GTGGTGGTGGTCBAAYTA | 95°C for 5 min; 40 cycles of 95°C for 30 s, 55°C for 30 s, 72°C for 30 s; 72°C for 5 min |  |
| CMX clade B *amoA* gene | CB377f | GTACTGGTGGGCBAAYTT |  |  |
| CMX clade A and B *amoA* gene | C576r | GAAGCCCATRTARTCNGCC |  |  |

# Supplementary Figures

## Fig. S1. Quantile function plot showing the electrical conductivity of lake/reservoirs and soils on a global scale. EC distributions were derived from global datasets for lake/reservoirs (filtered for non-coastal locations) and for soil (filtered for non-zero values) [19]. In the soil dataset, a large fraction of EC values below the median are derived from imputed data rather than direct measurements, reflecting the structure of the dataset [19]. Dashed lines indicate the electrical conductivity of the media used in this study. Details on the composition of MWM and AFW are provided in Supplementary Table S1.


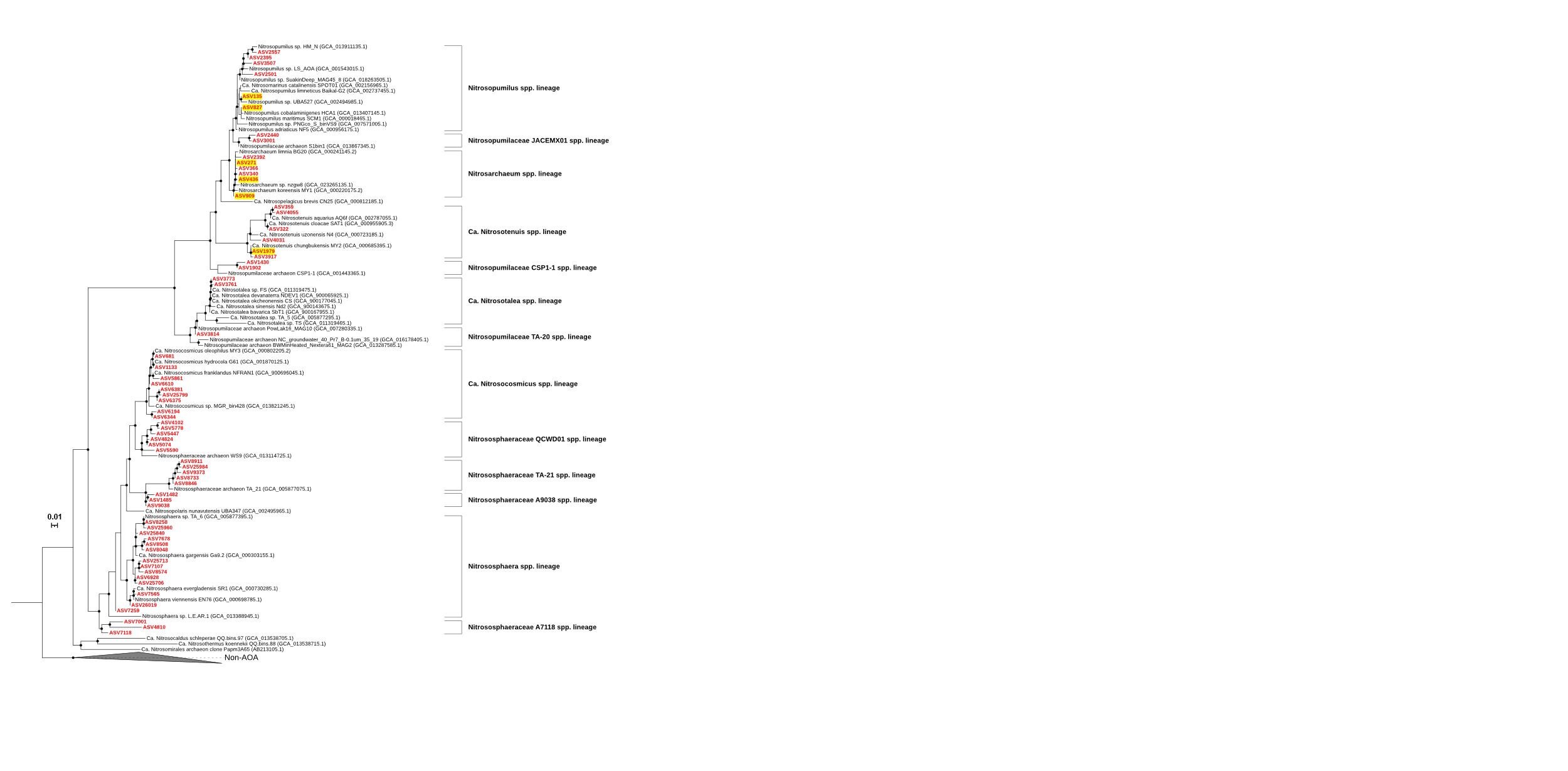


**A**


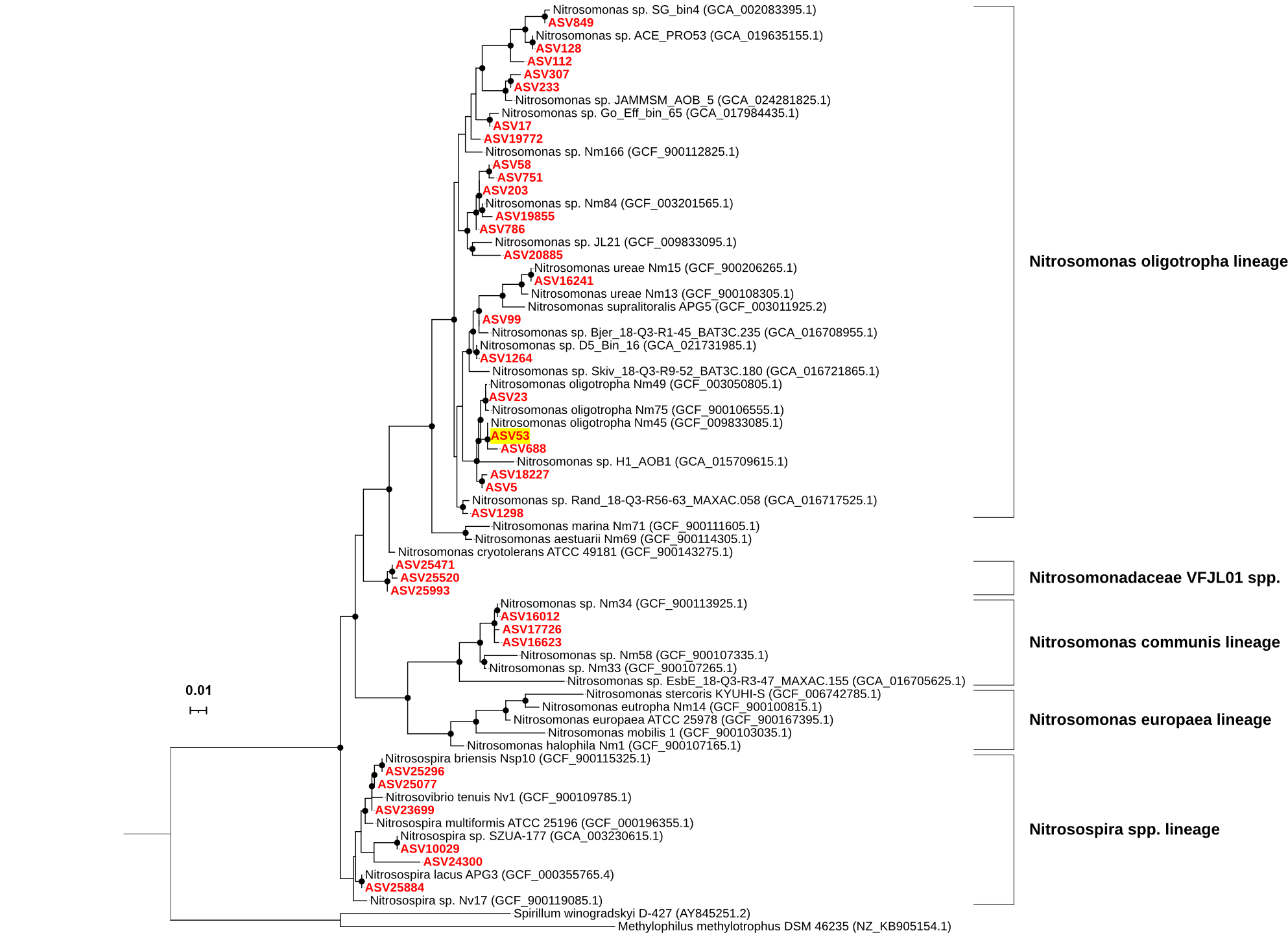


**B**


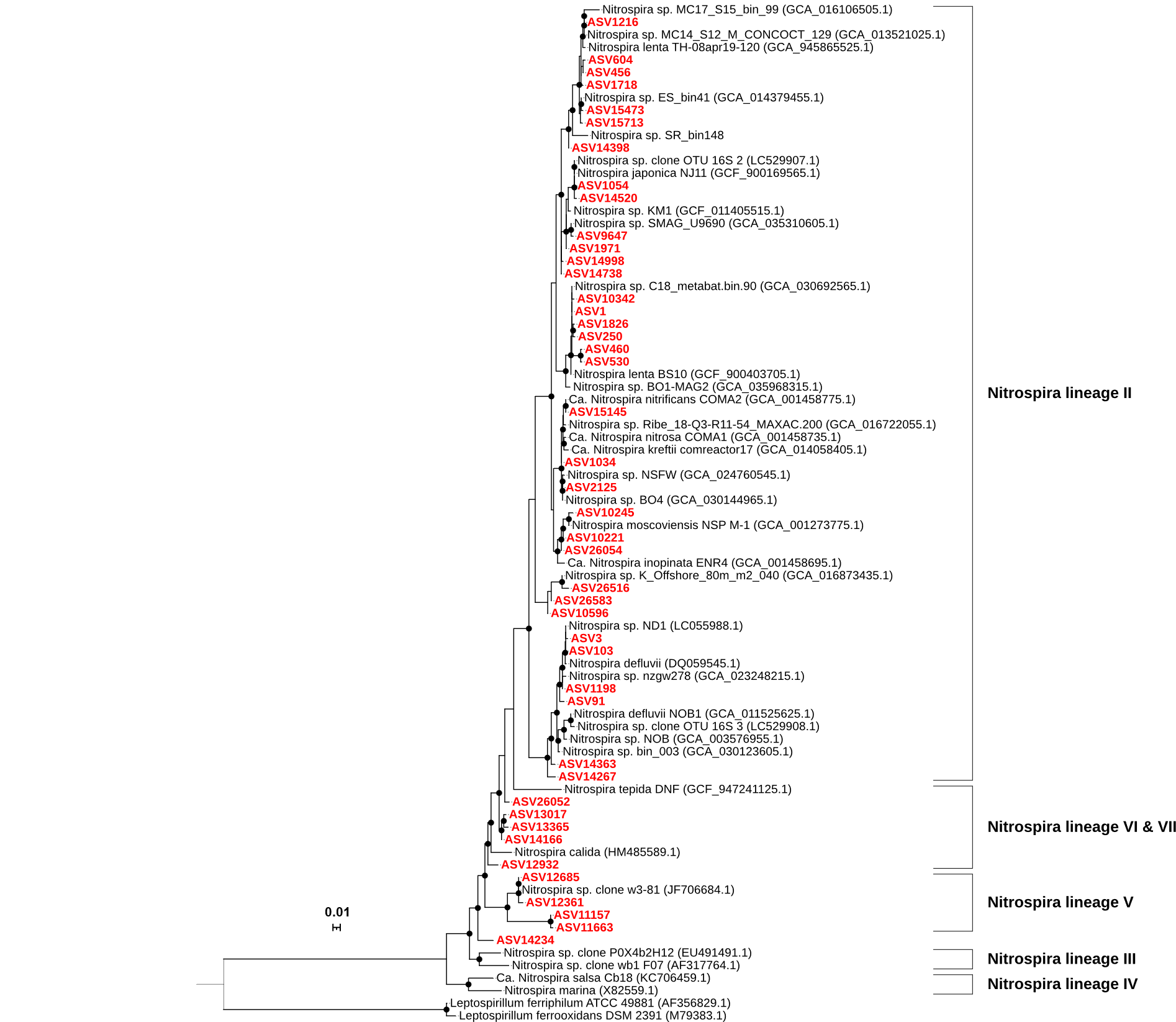


**C**

## Fig. S2. 16S rRNA gene-based phylogenetic trees of ammonia oxidizers and the genus *Nitrospira*. Phylogenetic trees were constructed based on 16S rRNA gene sequences corresponding to the ASVs shown in Figures 2 and 4. (A) ammonia-oxidizing archaea (AOA), (B) ammonia-oxidizing bacteria (AOB), (C) members of the genus *Nitrospira*. Black circles indicate nodes with ≥70% bootstrap support. ASV sequences are shown in red, and phylogenetic trees were inferred using the methods described in the Materials and Methods section. ASVs mentioned in the main text are highlighted in yellow. The models SYM+I+R4 (A), TPM3+I+R3 (B), and TIM3+F+I+R3 (C) were selected by ModelFinder [20] and used for tree inference.

## Fig. S3. Nitrate accumulation by *Nitrosarchaeum* *koreense* MY1 in Soyang reservoir microcosms. *N. koreensis* MY1 (~10^7^ cells ml^−1^) was inoculated into unfiltered water from the Soyang reservoir (81.2 μS/cm) under different salinity conditions: 0× (MWM without basic salts) and 10× MWM. ATU was added to inhibit bacterial ammonia oxidation. Nitrate accumulation was monitored over time. Under 10× MWM, nitrification proceeded efficiently, with complete ammonia oxidation within 26 days, whereas a significant delay was observed under 0× MWM. Error bars indicate standard deviations of biological replicates (*n* ≥ 3).

## Fig. S4. Growth of ammonia oxidizers under different salinity conditions. Specific growth rates of ammonia oxidizers, including the LD1 enrichment culture that was dominated by the *N. oligotropha* lineage, were determined under varying salinity conditions. The composition of MWM (0.5–128×) and AFW are detailed in Supplementary Table S1. Error bars represent standard deviations of biological replicates (*n* ≥ 3). Significant differences between treatments for each nitrifier are indicated by different letters (one-way ANOVA, Tukey’s test, *P* < 0.001).

## Fig. S5. No detection of AOA and comammox in the LD1 enrichment culture incubated under 1× and 10× MWM conditions. Gene copy numbers of ammonia oxidizers in the LD1 enrichment culture after oxidation of 0.1 mM ammonium under 1× and 10× MWM conditions. Only AOB *amoA* genes were detected, indicating no interference by other ammonia oxidizers. “initial” means before incubation. Dominance of the *Nitrosomonas oligotropha* lineage in the enrichment culture is shown in Supplementary Dataset S1. Error bars represent standard deviations of biological replicates (*n* ≥ 3). n.d., not detected.


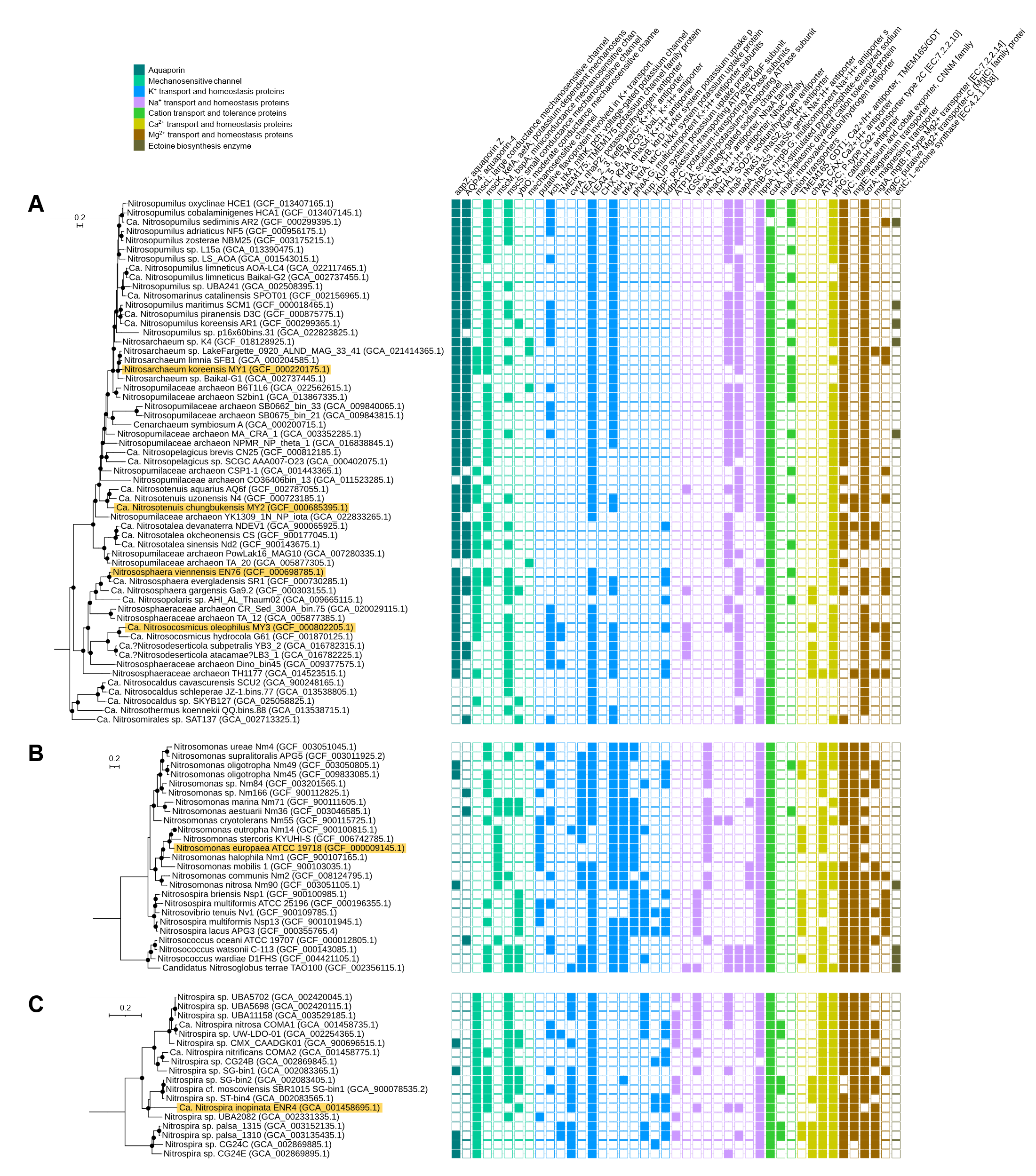


## Fig. S6. Comparative genomic repertoire of osmoregulatory systems in ammonia oxidizers. The phylogenomic tree includes representative genomes of (A) ammonia-oxidizing archaea (AOA; *n*= 57), (B) ammonia-oxidizing bacteria (AOB; *n*= 25), (C) comammox clade A (CMX-A; *n*= 14) and comammox clade B (CMX-B; *n*= 5). The complete dataset includes 463 genomes (see Supplementary Dataset S1). The tree was constructed using the Anvi’o phylogenomics workflow (see Materials and Methods for details). Black circles indicate nodes with ≥70% bootstrap support. Strains used in salinity-dependent growth experiments (see Fig. 5) are highlighted in yellow. Gene presence or absence was determined based on KEGG annotations. See the Materials and Methods section for details on gene selection and annotation procedures. Solid and open squares represent gene presence and absence, respectively.


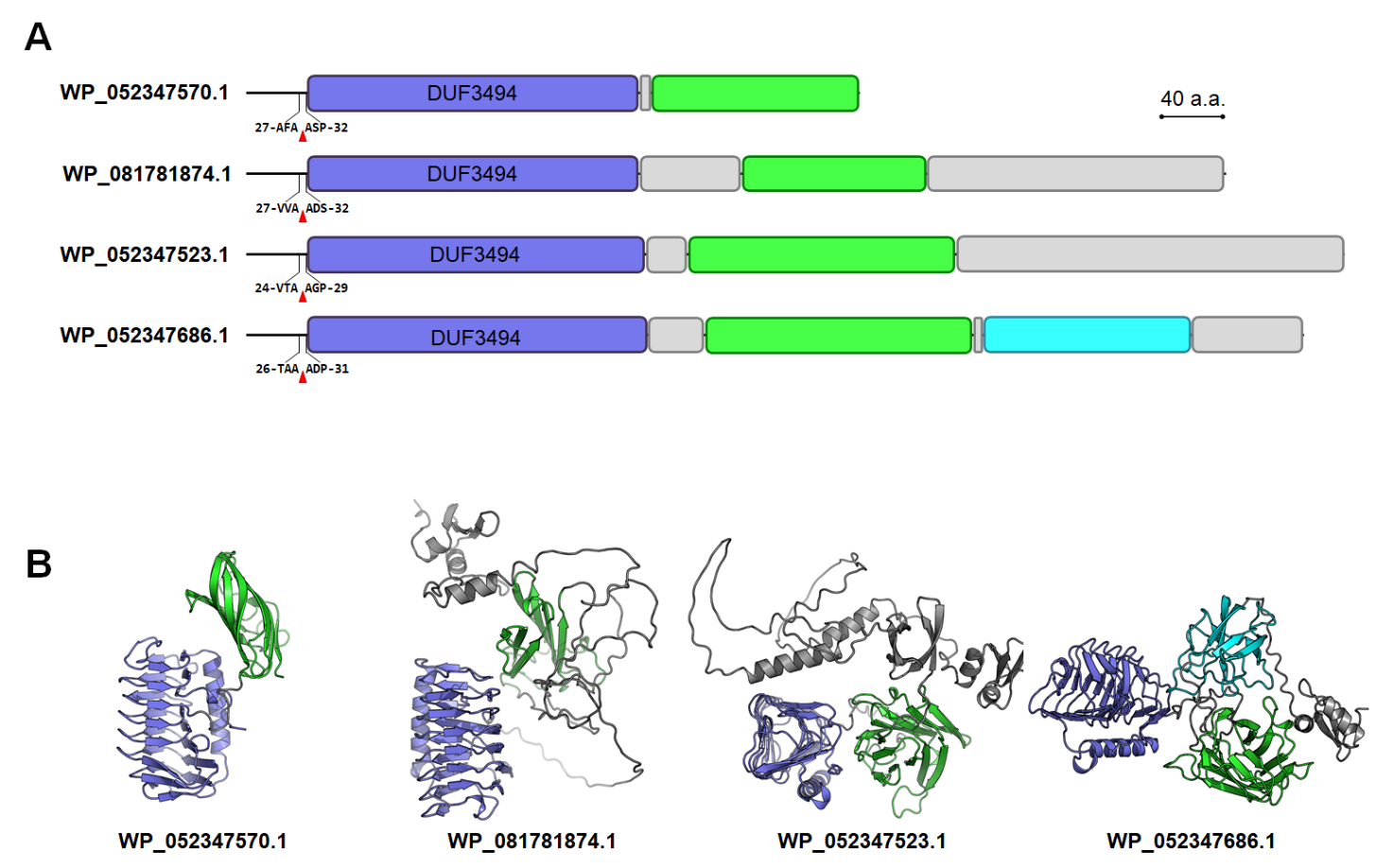


## Fig. S7. Sequence analysis and structural prediction of IBP-like proteins from “*Candidatus* Nitrosotenuis chungbukensis” MY2. (A) Signal peptide cleavage sites are indicated with red triangles. Schematic domain structures include DUF3494 domain (purple), β-sandwich domain (green), and β-barrel domain (cyan), and loop regions (grey). (B) Ribbon representations illustrate the overall structures of the mature proteins (excluding signal peptides). The color scheme used in A is applied to distinguish individual domains.

# Legends for Supplementary Datasets

**Dataset S1.** ASVs of ammonia oxidizers, including the *Nitrospira* lineage, amplified from soil slurry and freshwater microcosm samples.

**Dataset S2.** Genomic inventory of osmoregulatory genes in ammonia oxidizers.

**Dataset S3**. Transcriptome sequencing summary for the analyzed samples.

**Dataset S4.** DESeq2 analysis results based on raw count data from 16S rRNA gene amplicon sequencing of soil and freshwater microcosms at the genus level.

**Dataset S5.** Gene list and transcriptomic abundances in *Nitrososphaera viennensis* EN76 cells grown under 1× and 10× MWM conditions.

**Dataset S6.** Gene list and transcriptomic abundances in “*Candidatus* Nitrosotenuis chungbukensis” MY2 cells grown under 1× and 10× MWM conditions.

**Dataset S7.** Top 25 up- and down-regulated genes in *Nitrososphaera viennensis* EN76 cells grown under 1× and 10× MWM conditions (baseMean >10)^a^.

**Dataset S8.** Top 25 up- and down-regulated genes in “*Candidatus* Nitrosotenuis chungbukensis” MY2 cells grown under 1× and 10× MWM conditions (baseMean >10)^a^.

**Dataset S9.** Selected genomic functions of “*Candidatus* Nitrosotenuis chungbukensis” MY2 and *Nitrososphaera viennensis* EN76 with corresponding transcriptomic responses and proteomic detection under hypoosmotic conditions.

**Dataset S10.** Gene list and transcriptomic abundances in *Nitrosomonas europaea* ATCC 19718 cells grown under 1× and 10× MWM conditions.

# References

1. Wood JM. Osmosensing by bacteria: Signals and membrane-based sensors. *Microbiol Mol Biol Rev*. 1999;**63**:230-62 <https://doi.org/10.1128/mmbr.63.1.230-262.1999>

2. Zhang H, Pan Y, Hu L *et al.* TrkA undergoes a tetramer-to-dimer conversion to open TrkH which enables changes in membrane potential. *Nat Commun*. 2020;**11**:547 <https://doi.org/10.1038/s41467-019-14240-9>

3. Ding B, Zhang X, Xu Y *et al.* The bacterial potassium transporter gene MbtrkH improves K^+^ uptake in yeast and tobacco. *PLoS One*. 2020;**15**:e0236246 <https://doi.org/10.1371/journal.pone.0236246>

4. Epstein W. Chapter 9 The Kdp system: A bacterial K^+^ transport ATPase. In: Bronner F, Kleinzeller A (eds.). *Current Topics in Membranes and Transport*, Academic Press. 153-75. Retreived from <https://www.sciencedirect.com/science/article/pii/S0070216108601550>

5. Kumar S, Nicholas DJ. Na^+^ and K^+^ transport in *Nitrosomonas europaea* and *Nitrobacter agilis*. *Biochim Biophys Acta*. 1984;**765**:268-74 <https://doi.org/10.1016/0005-2728(84)90165-8>

6. Tanghe A, Van Dijck P, Thevelein JM. Why do microorganisms have aquaporins? *Trends Microbiol*. 2006;**14**:78-85 <https://doi.org/10.1016/j.tim.2005.12.001>

7. Tyerman SD, McGaughey SA, Qiu J *et al.* Adaptable and multifunctional ion-conducting aquaporins. *Annu Rev Plant Biol*. 2021;**72**:703-36 <https://doi.org/10.1146/annurev-arplant-081720-013608>

8. Tyerman SD, Niemietz CM, Bramley H. Plant aquaporins: multifunctional water and solute channels with expanding roles. *Plant, Cell & Environment*. 2002;**25**:173-94 <https://doi.org/https://doi.org/10.1046/j.0016-8025.2001.00791.x>

9. Tong H, Hu Q, Zhu L *et al.* Prokaryotic Aquaporins. *Cells*. 2019;**8**:1316 <https://doi.org/10.3390/cells8111316>

10. Wilson ME, Maksaev G, Haswell ES. MscS-like mechanosensitive channels in plants and microbes. *Biochemistry*. 2013;**52**:5708-22 <https://doi.org/10.1021/bi400804z>

11. Ramsey K, Britt M, Maramba J *et al.* The dynamic hypoosmotic response of *Vibrio cholerae* relies on the mechanosensitive channel MscS. *bioRxiv*. 2023 <https://doi.org/10.1101/2023.05.08.539864>

12. Sukharev SI, Sigurdson WJ, Kung C *et al.* Energetic and spatial parameters for gating of the bacterial large conductance mechanosensitive channel, MscL. *J Gen Physiol*. 1999;**113**:525-40 <https://doi.org/10.1085/jgp.113.4.525>

13. Moller E, Britt M, Schams A *et al.* Mechanosensitive channel MscS is critical for termination of the bacterial hypoosmotic permeability response. *J Gen Physiol*. 2023;**155** <https://doi.org/10.1085/jgp.202213168>

14. Mount J, Maksaev G, Summers BT *et al.* Structural basis for mechanotransduction in a potassium-dependent mechanosensitive ion channel. *Nat Commun*. 2022;**13**:6904 <https://doi.org/10.1038/s41467-022-34737-0>

15. Abby Sophie S, Kerou M, Schleper C. Ancestral reconstructions decipher major adaptations of ammonia-oxidizing archaea upon radiation into moderate terrestrial and marine environments. *mBio*. 2020;**11**:10.1128/mbio.02371-20 <https://doi.org/10.1128/mbio.02371-20>

16. Widdel F, Bak F. Gram-negative mesophilic sulfate-reducing bacteria. *The prokaryotes: a handbook on the biology of bacteria: ecophysiology, isolation, identification, applications*, Springer. 3352-78

17. Kwon H-H, An K-G. Trophic state index (TSI), spatial gradient characteristics and the empirical models for eutrophication evaluations in Daecheong reservoir. *J Environ Sci Int*. 2014;**23**:1537-49

18. An K-G, Park SS. Influence of seasonal monsoon on the trophic state deviation in an asian reservoir. *Water, Air, Soil Pollut*. 2003;**145**:267-87 <https://doi.org/10.1023/A:1023688819724>

19. Shangguan W, Dai Y, Duan Q *et al.* A global soil data set for earth system modeling. *J Adv Model Earth Syst*. 2014;**6**:249-63 <https://doi.org/10.1002/2013MS000293>

20. Kalyaanamoorthy S, Minh BQ, Wong TKF *et al.* ModelFinder: Fast model selection for accurate phylogenetic estimates. *Nat Methods*. 2017;**14**:587-89 <https://doi.org/10.1038/nmeth.4285>
